# Energetics of Formation and Fragmentation of Carbon-Centered Radicals α- to Nitrogen and Oxygen in *N*,*N*,*O*‑Trimethylhydroxylamine in Comparison to Trimethylamine and Dimethyl Ether: Implications for Medicinal and Synthetic Chemistry

**DOI:** 10.1021/acsphyschemau.6c00075

**Published:** 2026-08-08

**Authors:** R. Houston Givhan, Henry F. Schaefer, David Crich

**Affiliations:** † Department of Chemistry, 1355 University of Georgia, 302 East Campus Road, Athens, Georgia 30602, United States; ‡ Center for Computational Quantum Chemistry, University of Georgia, 1004 Cedar Street, Athens, Georgia 30602, United States; § Innovations in Drug Discovery Program, Department of Pharmaceutical and Biomedical Sciences, University of Georgia, 250 West Green Street, Athens, Georgia 30602, United States

**Keywords:** hydroxylamines, C–H bond dissociation energies, radical reactions, structural alerts, N–O bond cleavage, bioisosteres

## Abstract

α–C–H bond dissociation energies (BDEs) have been computed for *N*,*N*,*O*-trimethylhydroxylamine, trimethylamine, and dimethyl ether, leading to the conclusion that there is no advantage to hydrogen abstraction from trisubstituted hydroxylamines over the corresponding tertiary amines and ethers. Additionally, the computed vertical ionization potential for s, trimethylamine, and dimethyl ether, leading to the conclusion that there is no advantage to hydrogen abstraction from trisubstituted hydroxylamines over the corresponding tertiary amines and ethers. Additionally, the computed vertical ionization potential for *N*,*N*,*O*-trimethylhydroxylamine is greater than that for either trimethylamine or dimethyl ether. Together with the established N–O BDEs of ∼50 kcal.mol^–1^, these findings argue strongly against common notions of trisubstituted hydroxylamines as inherently chemically or metabolically unstable and refute their designation as red flags or structural alerts in drug discovery campaigns. Subsequent to hydrogen abstraction α- to either nitrogen or oxygen, hydroxylamines undergo facile exothermic N–O bond cleavage to give either O- or *N*-centered radicals. It is this radical-induced cleavage of hydroxylamines that synthetic chemists exploit for *N*- and O-centered radical generation rather than any perceived weakness of the N–O bond in the unactivated hydroxylamines themselves.

## Graphical abstract

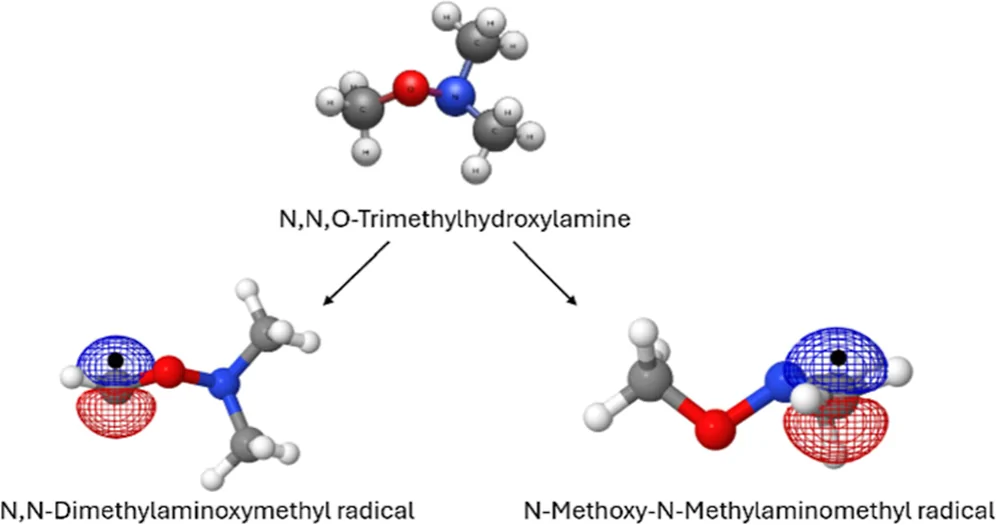

## Introduction

Hydroxylamines and their derivatives including the hydroxamic and thiohydroxamic acids are found in numerous natural products and play multiple and ever-expanding roles in both organic and medicinal chemistry.^1–4^ Applications in the synthetic organic chemical field date to Barton’s use of nitroso esters as photolytic sources of alkoxy radicals for remote functionalization,^5,6^ through Barton’s use of *O*-acyl thiohydroxamates in decarboxylation reactions,^7,8^ to the various uses of *N*-acyloxyphthalimides as radical precursors pioneered by Okada^9^ and popularized by the Overman and MacMillan groups,^10,11^ to the largely parallel uses of *O*-acyl oximes originating from the Walton laboratory.^12^ Beyond radical chemistry, *O*-acyl derivatives of hydroxylamines have served widely as nitrogen-centered electrophiles, both with and without transition metal catalysis.^13–19^ In bioorganic and medicinal chemistry, hydroxylamines find widespread use in neoglycoconjugation^17,20–27^ and are fundamental elements of the isoxazolidine class of preferred pharmacophores.^28^ Additionally, the hydroxamic acid moiety has been widely applied as a siderophore and more recently as a key element of HDAc inhibitors.^4^

Many of the applications of hydroxylamines and (thio)­hydroxamic acids in synthetic organic chemistry rely on the perceived weakness of the N–O bond, while in the era of high-throughput screening in medicinal chemistry, the very presence of a N–O bond (except in heterocycles) is often considered a structural alert or red flag.^29–35^ Exceptions to this latter rule typically result from more traditional medicinal chemistry programs.^36–40^ The consideration of hydroxylamines as structural alerts has its roots in the metabolic activation of the mono- and disubstituted variants to genotoxic and/or mutagenic electrophilic species^41–44^ and more broadly in the perception of heteroatom–heteroatom bonds as being weak and reactive.

We have argued that the broad moratorium on the use of hydroxylamines in medicinal chemistry should not apply to the fully substituted *N*,*N*,*O*-trialkyl hydroxylamines as, in the absence of either a NH or an OH bond, they are not susceptible to the metabolic pathways that convert the lesser substituted variants to mutagens.^45–54^ Moreover, owing to their low basicity and polarity, the *N*,*N*,*O*-trialkyl hydroxylamines are very weak electrophiles that are compatible with a wide variety of reaction conditions. Finally, in the absence of conjugation, the *N*,*N*,*O*-trialkyl hydroxylamines are colorless and not susceptible to activation by UV/visible light and, with N–O bond dissociation energies (BDEs) around 54 to 61 kcal mol,^55,56^ they are stable to temperatures well in excess of the ambient. Indeed, in our studies on the applications of *N*,*N*,*O*-trialkyl hydroxylamines as minimal bioisosteres in medicinal chemistry, we find that (i) they are stable to a wide range of oxidative and reductive processes and other chemical reactions (Table 1), (ii) typically are not genotoxic in the Ames assay, (iii) in multiple instances convey enhanced stability toward liver microsomes and hepatocytes compared to isosteric tertiary centers, ethers and tertiary amines, and (iv) can be used to improve other pharmacokinetic parameters such as cell membrane and blood brain barrier penetration,^45–54^ as has also been reported for the oxyguanidine unit in the nonproteinogenic amino acid canavanine.^39,40^

**Table 1:** Reactions Successfully Conducted in the Presence of *N*,*N*,*O*-Trisubstituted Hydroxylamines in the Authors’ Laboratories

reaction type	examples
oxidations	• DDQ oxidations • periodate diol cleavage • Pinnick **•** Parekh **•** ozonolysis
reductions	• reductive amination • Et_3_SiH/BF_3_ reductions • exposure to H_2_S and thiols • exposure to lithium and magnesium amides • Staudinger reaction with PMe_3_ • LiAlH_4_ reductions • controlled hydrogenolysis of Cbz groups
miscellaneous	• HCl or TFA Boc removal • BBr_3_ and BCl_3_ benzyl ether cleavage • desilylation with fluoride • glycosylation • alcohol activation with DAST • amide formation • exposure to SOCl_2_ • exposure to azide and phenate anions • Suzuki reaction
	• Buchwald–Hartwig reaction • hydrazinolysis of phthalimides • exposure to acidic and basic resins • Ullmann–Goldberg reaction

To allay concerns of the metabolic instability of trisubstituted hydroxylamines, we have conducted a high-level computational study of the bond dissociation energies of the C–H bonds α- to both nitrogen and oxygen in *N*,*N*,*O*-trimethylhydroxylamine **1** in comparison to both dimethyl ether **2** and trimethylamine **3**. To shed light on the radical-induced fragmentation of hydroxylamine N–O bonds of interest to both medicinal and synthetic organic chemists, we have also studied and report on the fragmentation of the above radicals. Overall, we find that trisubstituted hydroxylamines are no more susceptible to α-hydrogen abstraction or ionization than the corresponding ethers and amines and so no more likely to undergo oxidative metabolism. We further report that, while the N–O BDEs of simple hydroxylamines preclude homolytic cleavage under ambient and synthetically useful conditions, fragmentation of the N–O is rapid and exothermic following α-hydrogen abstraction.

## Results

We computed the structure of *N*,*N*,*O*-trimethylhydroxylamine **1** at the CCSD­(T)-pvTZ level of theory.^57,58^ Consistent with earlier work on hydroxylamine structure,^59,60^ X-ray crystallographic studies of other trisubstituted hydroxylamines,^47,50^ the recent computations of Bach and Schlegel,^56^ and our own calculations on more complex trisubstituted hydroxylamines,^51^ the molecule adopts a conformation in which the O–Me bond eclipses the lone pair on nitrogen, while the two *N*–Me bonds eclipse the two lone pairs on oxygen, overall minimizing steric and electrostatic repulsions. The structures of dimethyl ether **2** and trimethylamine **3** were computed by the same method for comparison purposes (Figure 1). Key bond lengths and bond angles for **1**, **2**, and **3** are provided in Figure 1, while complete tables of bond lengths and bond and torsion angles are presented in the Supporting Information.

**Figure 1:**
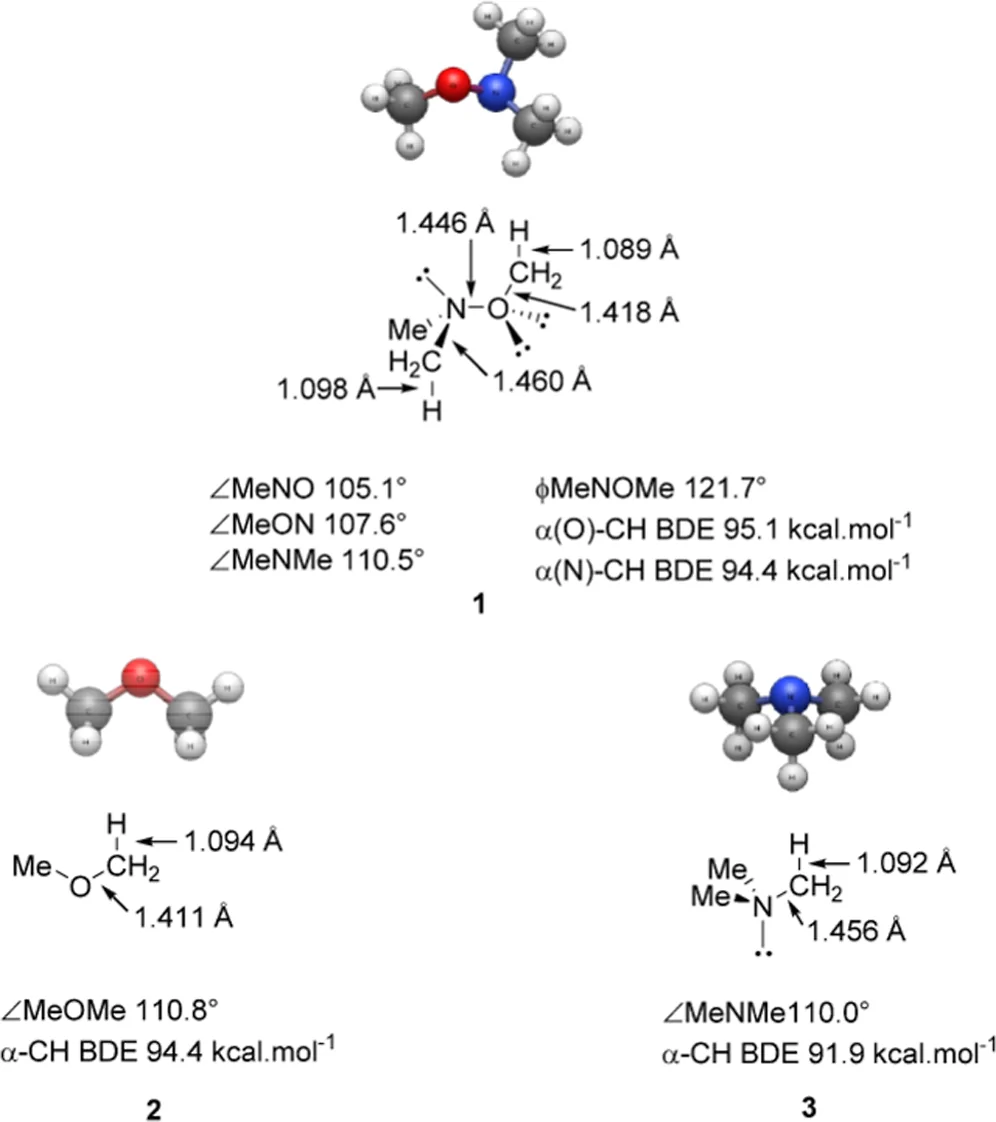
Key computed bond lengths, bond and torsion angles, and α–C–H bond dissociation energies in *N*,*N*,*O*-trimethylhydroxylamine **1**, dimethyl ether **2**, and trimethylamine **3**.

We next computed at the same level of theory the structures of the radicals **4** and **5** arising from abstraction of hydrogen from C–H bonds α- to oxygen and nitrogen in **1**, respectively, and for comparison purposes of the methoxymethyl **6** and dimethylaminomethyl radicals **7**, with key bond lengths, angles, and torsion angles presented in Figure 2 and complete lists in the Supporting Information. Consistent with the rehybridization from sp^3^ to sp^2^ at carbon on hydrogen atom abstraction, in every case, the bond angles around the radical center in **4** and **5** open up with respect to the parent closed shell system **1** and the remaining bonds to the unsaturated carbon are shortened. This contraction of the C–O bond in **4** and of the C–N bond in **5** on radical formation at carbon is significant, is consistent with delocalization of the unpaired electron onto the adjacent heteroatom, and matches that seen in the corresponding ether and amine-derived radicals **6** and **7**.^61^ Overall, there is nothing unusual about the structure of hydroxylamine-derived radicals **4** and **5** in comparison to the ether and amine congeners **6** and **7**.

**Figure 2:**
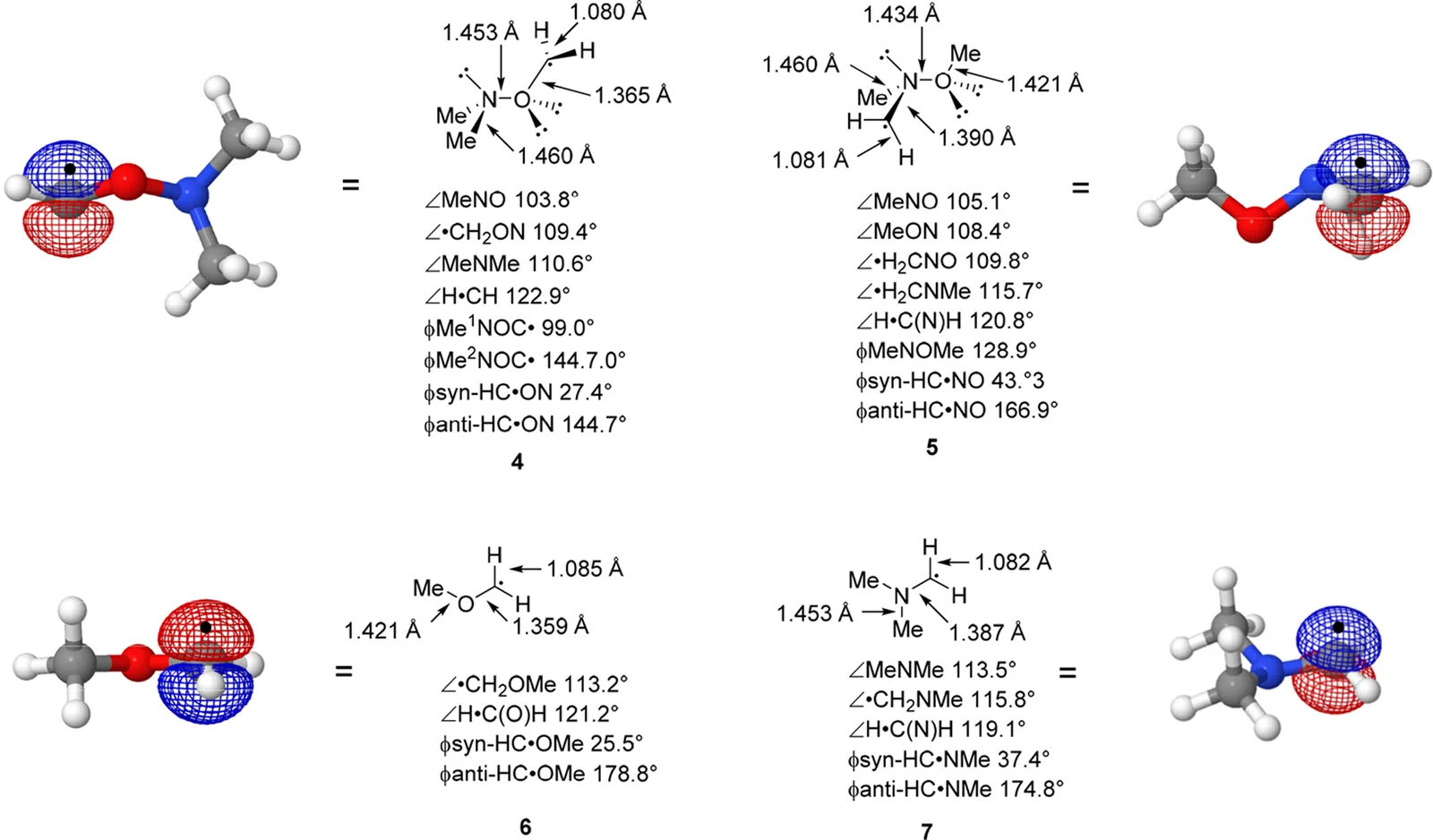
Key computed bond lengths, bond angles, and torsion angles in *N*,*N*-dimethylaminoxymethyl **4**, *N*-methoxy-*N*-methylaminomethyl **5**, dimethylaminomethoxymethyl **6**, and dimethylaminomethyl **7** radicals.

Next, we computed the C–H BDEs in the methyl groups of *N*,*N*,*O*-trimethylhydroxylamine **1**, dimethyl ether **2**, and trimethylamine **3** at the CCSD­(T)-pvTZ level of theory.^57,58^ The C–H bond adjacent to oxygen in **1** was found to have a BDE of 95.1 kcal mol^–1^, while the corresponding C–H bond adjacent to nitrogen had a BDE of 94.4 kcal mol^–1^ (Figure 1). BDEs for hydrogen atom abstraction from dimethyl ether **2** and trimethylamine **3** were found to be 94.4 and 91.9 kcal mol^–1^, respectively, both of which are in good agreement with experimental values^55^ and so serve to benchmark the computations. Overall, the C–H bonds adjacent to oxygen in hydroxylamine **1** have the same BDE as those in dimethyl ether, while those adjacent to nitrogen in **1** are marginally (2.5 kcal mol^–1^) stronger than those in trimethylamine: there is no compelling thermodynamic advantage for hydrogen atom abstraction adjacent to either oxygen or nitrogen in hydroxylamine **1** compared to the corresponding ether **2** and amines **3**.

To estimate the relative kinetics of hydrogen atom abstraction α- to either oxygen or nitrogen in hydroxylamine **1**, we employed the hydroxy radical as a model for the biologically relevant oxidants such as cytochromes and hydroxyl radicals. Both hydrogen atom abstractions were found to be essentially barrierless (Figure 3), consistent with computational barriers for hydrogen atom abstraction by the hydroxyl radical from dimethyl ether and trimethylamine at comparable levels of theory^62,63^ and with the reported second order rate constant for hydrogen abstraction from trimethylhydroxylamine **1** by the benzophenone triplet in benzene (1.8 × 10^8^ M^–1^ s^–1^).^64^ The evidence suggests, therefore, that while hydrogen abstraction from C–H bonds α- to oxygen or nitrogen in trisubstituted hydroxylamines by highly electrophilic radicals is rapid, it is no more so than in the corresponding ethers or trisubstituted amines.

**Figure 3:**
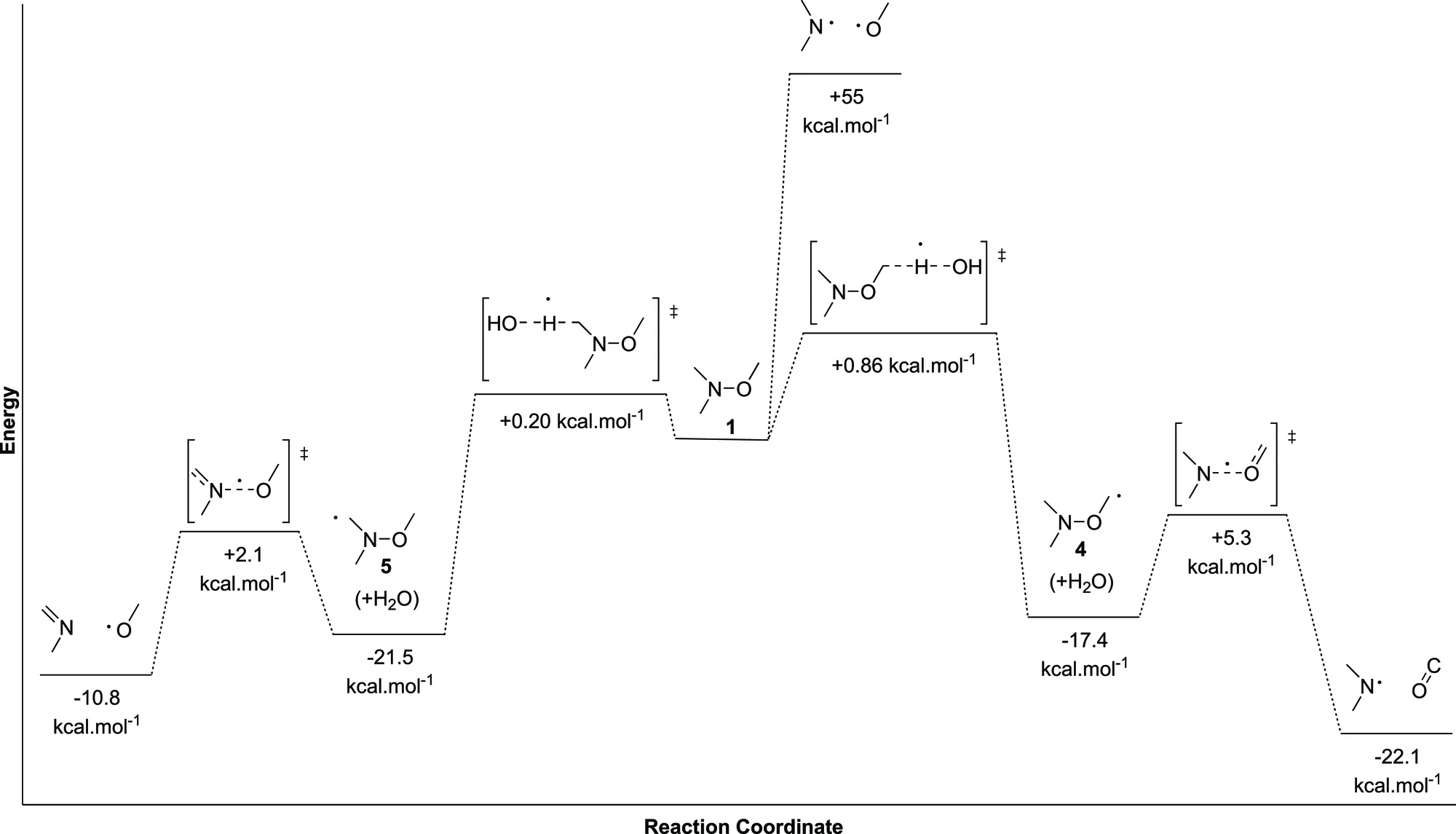
Activation energies and exothermicities for hydrogen atom abstraction by the hydroxy radical α- to both O- and *N*- in hydroxylamine **1** and for the subsequent fragmentation reactions compared to the endothermic homolytic cleavage of the N–O bond (not to scale).

Next, considering the possibility that metabolic hydroxylamine oxidation may also proceed by single electron transfer to give an initial radical cation, we computed the ionization potentials (IPs) for hydroxylamine **1** and the comparators dimethyl ether **2** and trimethylamine **3** (Figure 4). The IPs determined are vertical excitations, taking the energy difference of the radical cations at CCSD­(T)/cc-pvTZ with respect to the neutral substrates at the same geometry. The NIST experimental values for **2** and **3** are also provided in Figure 4 and serve to benchmark the computations. Notably, the IP for the hydroxylamine **1** is approximately 1 eV greater than that of dimethyl ether and 2.5 eV greater than that of trimethylamine. Gas-phase photoelectron spectroscopy studies have previously placed the IPs for removal of an electron from the nitrogen (n_σ_) lone pair, oxygen (n_π_) lone pair, and either the oxygen (n_σ_) lone pair or the N–O σ-bond of **1** at 8.81, 9.93, and 12.51 eV, respectively.^65^ The computed IPs argue against the possibility that trisubstituted hydroxylamines suffer metabolism by single-electron transfer more rapidly than the corresponding ethers or trisubstituted amines.

**Figure 4:**
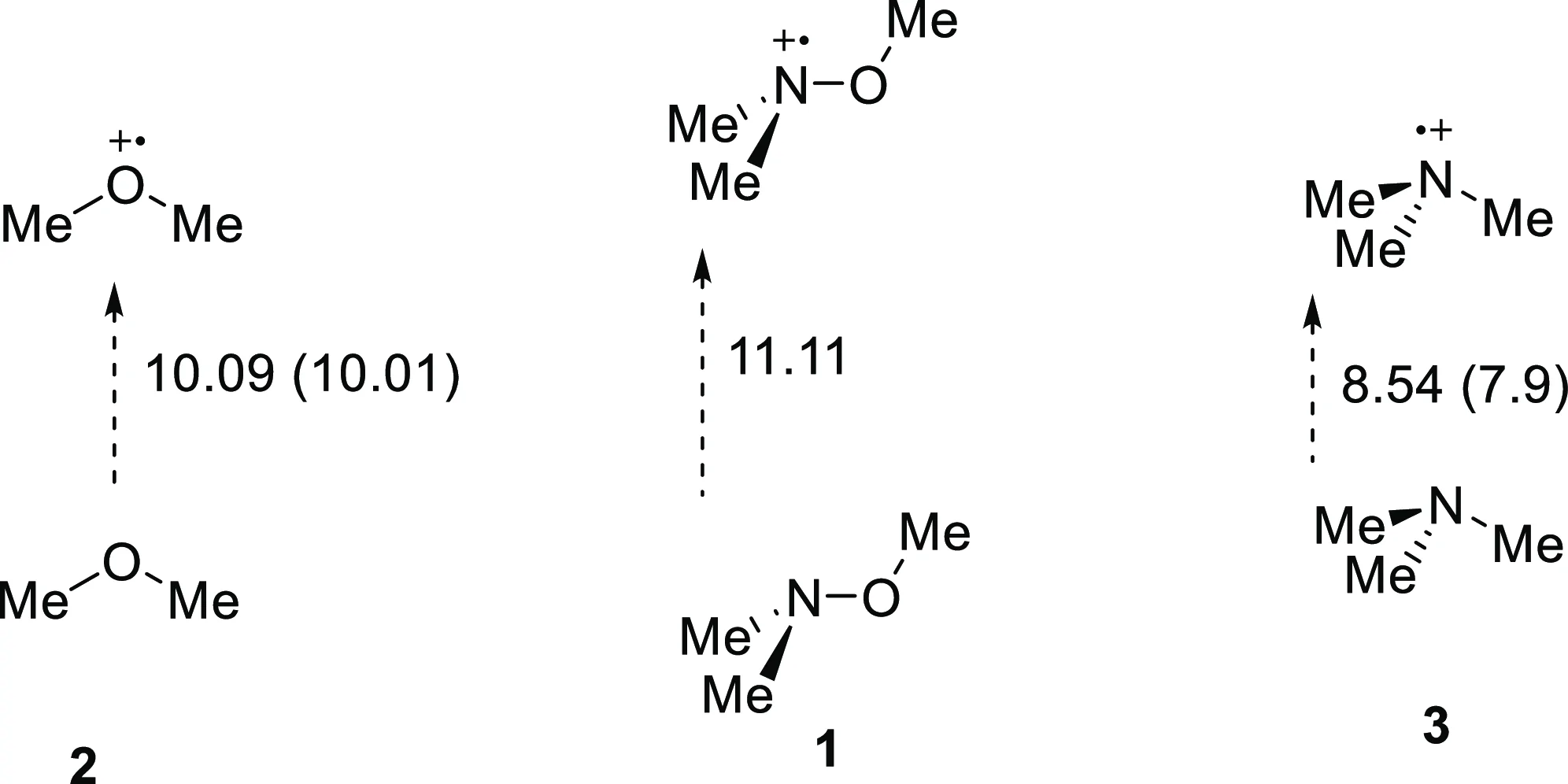
Computed ionization potentials (eV) of hydroxylamine 1, dimethyl ether, and trimethylamine and (in parentheses) NIST experimental values for dimethyl ether and trimethylamine.

Finally, we investigated the fragmentation of radicals **4** and **5** by N–O bond cleavage. Both fragmentations were exothermic and proceeded with minimal activation barriers indicative of rapid reaction at or below room temperature (Figure 3). The ease and exothermicity of these fragmentations stands in contrast to the highly endothermic homolysis of the N–O bond in **1** itself and is due to the weakening of the N–O bond by the adjacent radical, which is a broadly general phenomenon.^66,67^ Despite the similar energetics of fragmentation of **4** and **5**, that of dimethylaminoxymethyl radical **4** to formaldehyde and the dimethylamino radical was the more exothermic of the two by approximately 9 kcal mol^–1^, which can be understood in terms of the greater stability of the dimethylamino radical (BDE Me_2_N–H = 94.6 kcal mol^–1^)^55^ compared to the methoxy radical (BDE MeO–H = 104.2 kcal mol^–1^).^55^ On the other hand, the activation barrier for the fragmentation of **5** was lower than that of **4** by approximately 3 kcal mol^–1^ (Figure 3), which we interpret as arising from the polar nature^68–75^ of the transition state for fragmentation, which is in turn better accommodated in **5** than in **4** (Figure 5).

**Figure 5:**
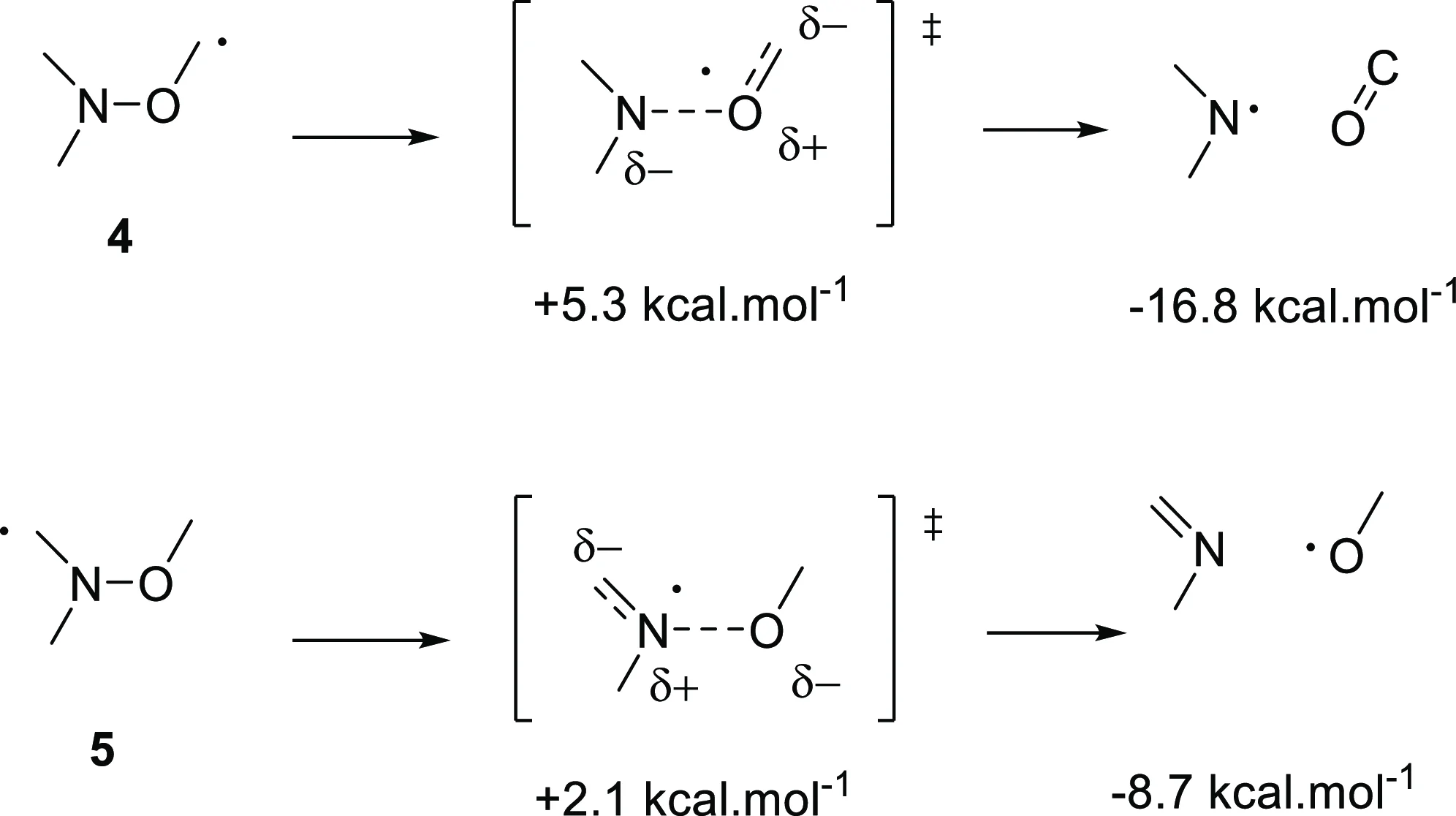
Polarized transition states for the fragmentation of **4** and **5**.

## Conclusion

The BDEs of C–H bonds α- to either nitrogen or oxygen in *N*,*N*,*O*-trimethylhydroxylamine are comparable to those in trimethylamine or dimethyl ether. Abstraction of hydrogen by hydroxyl radicals α- to either nitrogen or oxygen in *N*,*N*,*O*-trimethylhydroxylamine provides the corresponding radicals **4** and **5** whose structures closely resemble those of the radicals **6** and **7** derived by hydrogen abstraction from trimethylamine and dimethyl ether, respectively. The vertical ionization energy of trimethylhydroxylamine **1** is greater than that of either trimethylamine or dimethyl ether. Overall, neither the α–C–H BDEs of *N*,*N*,*O*-trimethylhydroxylamine, nor the structures of the corresponding radicals, nor the vertical ionization potential of *N*,*N*,*O*-trimethylhydroxylamine to the corresponding radical cation provide any support for the often-assumed preferential oxidative metabolism of trialkylhydroxylamines over the corresponding trialkylamines or dialkyl ethers. The designation of trialkylhydroxylamines as structural alerts or red flags in drug discovery campaigns, as compared to simple amines and ethers, has no basis in theory, consistent with recent experimental work from our laboratories.

Radicals **4** and **5** derived by hydrogen atom abstraction adjacent to nitrogen or oxygen in *N*,*N*,*O*-trisubstituted hydroxylamines undergo exothermic N–O bond cleavage with minimal activation barriers, owing to the weakening the scissile bond by the adjacent radical. The aminyl radicals (R_2_N^•^ and especially alkoxy radicals (RO^•^) generated in such radical-induced fragmentation of hydroxylamine N–O bonds are electrophilic species that are themselves capable of hydrogen atom abstraction from further C–H bonds and so of propagating the initial damage due to hydrogen atom abstraction. But this is a feature of radicals in general, and of reactive oxygen species (ROS) in particular, and one that is associated with many forms of metabolism and catabolism, and it is an indirect consequence of the mode of action of many therapeutic agents.^76–79^ Moreover, given that hydrogen atom abstraction α- to either nitrogen or oxygen necessarily precedes fragmentation to give either radicals aminyl or alkoxy radicals, and that this initial abstraction is no more facile than from corresponding amines and ethers, there is no reason to consider *N*,*N*,*O*-trisubstituted hydroxylamines as unusually facile sources of ROS or other radicals.

The same facile cleavage of N–O bonds following formation of radicals on adjacent atoms explains the utility of hydroxylamine derivatives as radical precursors in synthetic organic chemistry rather any inherent weakness of the hydroxylamine N–O bond itself. This principle holds regardless of whether the radical is generated by hydrogen atom abstraction or other means such as carbonyl group reduction as in Leonori’s electrophore concept,^17^ or radical addition to conjugated π-bond as in the Barton decarboxylation reaction.

## Experimental Section

All structures were optimized at the CCSD­(T)/cc-pVTZ level of theory. Harmonic vibrational frequencies were calculated by finite difference of energies at the same level of theory to determine zero-point vibrational energy (ZPVE) corrections with Molpro.^80–83^ Anharmonics were predicted with B3LYP/cc-pVTZ to give aZPVE corrections.^84^ Transition-state optimizations and intrinsic reaction coordinates (IRC) were carried out to determine the transition states as well as their connected reactant and products for hydroxyl radical α–C–H abstraction of the two radical formation pathways of *N*,*N*,*O*-trimethylhydroxylamine and the following radical fragmentation of the N–O bond at the B3LYP/def2-TZVP level of theory.^85^ The anharmonics and IRCs were computed using Orca 6.1.^86^ CCSDT^87^ and CCSDT­(Q)^88^ single-point energy corrections were also computed at cc-pVDZ with MRCC to accurately identify the bond dissociation energies.^89,90^

The BDEs are computed using a focal point approach developed by Allen and co-workers to determine bond strengths by comparing the relative energies of the neutral species with respect to their radical species plus hydrogen.^91,92^ Single-point energies at Hartree–Fock (HF), Møller–Plesset second-order perturbation theory (MP2), Coupled Cluster with singles and doubles (CCSD), and Coupled Cluster with singles, doubles, and perturbative triples CCSD­(T) values were computed up to basis set cc-pV5Z, while CCSDT and CCSDT­(Q) were computed with cc-pVDZ. These energies are then extrapolated to the complete basis set (CBS) limit using a three-point fitting equation for HF (eq 1) and a two-point fitting equation for correlation energies (eq 2).^93,94^

1 $$E_{HF}=A+Be^{-CX}$$

2 $$E_{Corr}=A+BX^{-3}$$

Equation 3 shows the total energy expression that will be representative of the bond dissociation energies of the α–C–H for the structures of *N*,*N*,*O*-trimethylhydroxylamine, trimethylamine, and dimethyl ether as well as the radical fragmentation of *N*,*N*,*O*-trimethylhydroxylamine radicals.

3 $$\Delta E=E_{products}-E_{reactant=}\delta E_{CCSDT/CBS}+\delta E_{ZPVE}+\delta E_{aZPVE}$$

## Supplementary Material



## Notes

Cartesian coordinates, bond lengths, bond angles, and bond dihedrals for all molecules, along with focal point tables for BDEs and radical fragmentation (PDF)
