# Supplementary material for: Energetics of Formation and Fragmentation of Carbon-Centered Radicals α- to Nitrogen and Oxygen in N,N,O‑Trimethylhydroxylamine in Comparison to Trimethylamine and Dimethyl Ether: Implications for Medicinal and Synthetic Chemistry
Source: ACS Phys Chem Au. 2026 Aug 8;6(5):1244–51. doi: 10.1021/acsphyschemau.6c00075 (PMC13614165; doi:10.1021/acsphyschemau.6c00075)
Supplement: Supplementary file 1 [file pg6c00075_si_001.pdf]

# Energetics of Formation and Fragmentation of Carbon-Centered Radicals α- to Nitrogen and Oxygen in *N*,*N*,*O*-Trimethylhydroxylamine in Comparison to Trimethylamine and Dimethyl Ether. Implications for Medicinal and Synthetic Chemistry

## Supporting Information

R. Houston Givhan,[a,b] Henry F. Schaefer III[a,b] and David Crich[a,c,*]

a) Department of Chemistry, University of Georgia, 302 East Campus Road, Athens, GA 30602, USA.

b) Center for Computational Quantum Chemistry, University of Georgia, 1004 Cedar Street, Athens, GA 30602, USA.

c) Innovations in Drug Discovery Program, Department of Pharmaceutical and Biomedical Sciences, University of Georgia, 250 West Green Street, Athens, GA 30602, USA.

Email: David.Crich@uga.edu

# Table of Contents

S1. N,N,O-Trimethylhydroxylamine
S2. N-Methoxy-N-methylaminomethyl radical
S3. N,N-Dimethylaminoxymethyl radical
S4. Dimethyl Ether
S5. Dimethylaminomethoxymethyl radical
S6. Trimethylamine
S7. Dimethylaminomethyl radical
S8. N,N,O-Trimethylhydroxylamine + Hydroxy radical
S9. N,N,O-Trimethylhydroxylamine + Hydroxy radical TS
S10. N-Methoxy-N-methylaminomethyl radical + water
S11. N-Methoxy-N-methylaminomethyl radical fragmentation TS
S12. N-methylmethanimine
S13. Methoxy radical
S14. N,N,O-Trimethylhydroxylamine + Hydroxy radical
S15. N,N,O-Trimethylhydroxylamine + Hydroxy radical TS
S16. N,N-Dimethylaminoxymethyl radical + water
S17. N,N-Dimethylaminoxymethyl radical fragmentation TS
S18. Dimethylamino radical
S19. Formaldehyde
S20. Focal point table of (CH3)2NOCH2• + H with respect to (CH3)2NOCH3.
S21. Focal point table of (CH3)(CH2•)NOCH3 + H with respect to (CH3)2NOCH3.
S22. Focal point table of methoxymethyl radical + H with respect to dimethyl ether.
S23. Focal point table of dimethylaminomethyl radical + H with respect to trimethylamine.
S24. Focal point table of dimethylaminyl radical + formaldehyde with respect to (CH3)2NOCH2•.
S25. Focal point table of N-Methylmethanimine + methoxyl radical with respect to (CH3)(CH2•)NOCH3.

# S1. N,N,O-trimethylhydroxylamine

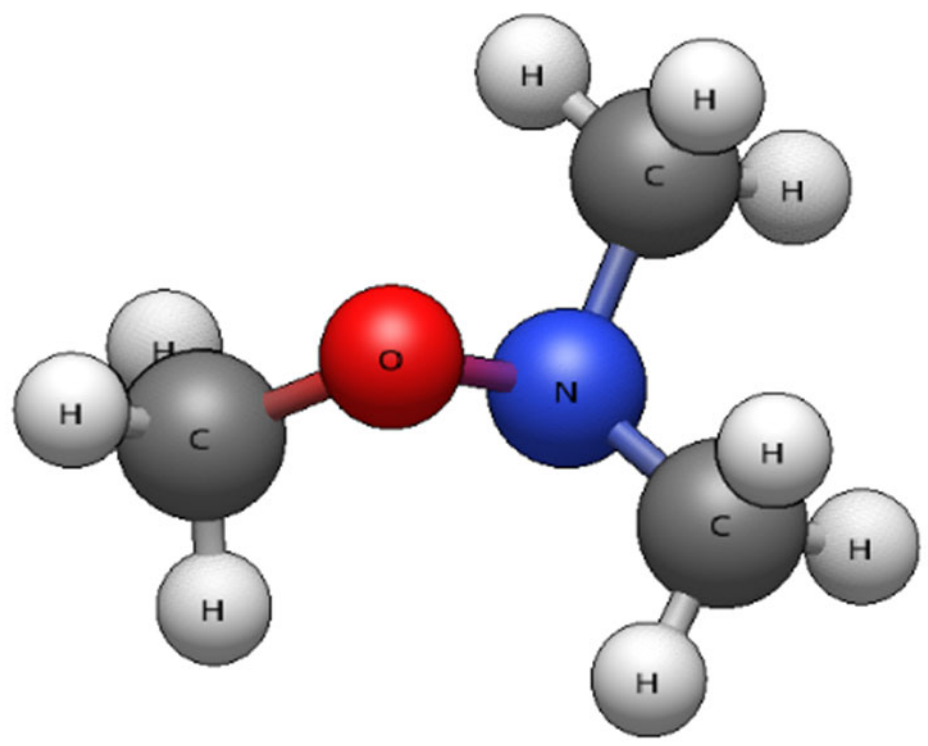


## Cartesian Coordinates

| # | Atom | X (Å) | Y (Å) | Z (Å) |
| --- | --- | --- | --- | --- |
| 1 | C1 | 1.154233 | 1.199674 | 0.001919 |
| 2 | H2 | 2.053151 | 1.231174 | -0.616590 |
| 3 | H3 | 1.440112 | 1.210322 | 1.062011 |
| 4 | H4 | 0.547177 | 2.078208 | -0.213621 |
| 5 | N5 | 0.398610 | -0.000008 | -0.346630 |
| 6 | C6 | 1.154181 | -1.199723 | 0.001921 |
| 7 | O7 | -0.740048 | 0.000017 | 0.545352 |
| 8 | H8 | -2.750713 | 0.000058 | 0.441508 |
| 9 | H9 | 1.440057 | -1.210383 | 1.062013 |
| 10 | H10 | 0.547087 | -2.078231 | -0.213621 |
| 11 | H11 | 2.053098 | -1.231261 | -0.616587 |
| 12 | H12 | -1.959274 | 0.890386 | -0.887824 |
| 13 | C13 | -1.910665 | 0.000040 | -0.254133 |
| 14 | H14 | -1.959311 | -0.890308 | -0.887820 |

### Bond Lengths

| # | Bond | Length |
| --- | --- | --- |
| 1 | C1 – H2 | **1.0916 Å** |
| 2 | C1 – H3 | **1.0980 Å** |

| # | Bond | Length |
|---|---|---|
| 3 | C1 – H4 | **1.0894 Å** |
| 4 | C1 – N5 | **1.4600 Å** |
| 5 | N5 – C6 | **1.4600 Å** |
| 6 | N5 – O7 | **1.4464 Å** |
| 7 | C6 – H9 | **1.0980 Å** |
| 8 | C6 – H10 | **1.0894 Å** |
| 9 | C6 – H11 | **1.0916 Å** |
| 10 | O7 – C13 | **1.4176 Å** |
| 11 | H8 – C13 | **1.0907 Å** |
| 12 | H12 – C13 | **1.0939 Å** |
| 13 | C13 – H14 | **1.0939 Å** |

## Bond Angles

B is the vertex atom in each A–B–C triple.

| # | Angle | Value |
|---|---|---|
| 1 | H2 – C1 – H3 | **109.41 °** |
| 2 | H2 – C1 – H4 | **108.87 °** |
| 3 | H2 – C1 – N5 | **108.34 °** |
| 4 | H3 – C1 – H4 | **109.16 °** |
| 5 | H3 – C1 – N5 | **111.91 °** |
| 6 | H4 – C1 – N5 | **109.09 °** |
| 7 | C1 – N5 – C6 | **110.51 °** |
| 8 | C1 – N5 – O7 | **105.08 °** |
| 9 | C6 – N5 – O7 | **105.08 °** |
| 10 | N5 – C6 – H9 | **111.91 °** |
| 11 | N5 – C6 – H10 | **109.09 °** |
| 12 | N5 – C6 – H11 | **108.34 °** |
| 13 | H9 – C6 – H10 | **109.16 °** |
| 14 | H9 – C6 – H11 | **109.41 °** |
| 15 | H10 – C6 – H11 | **108.87 °** |
| 16 | N5 – O7 – C13 | **107.59 °** |
| 17 | O7 – C13 – H8 | **106.04 °** |
| 18 | O7 – C13 – H12 | **111.31 °** |
| 19 | O7 – C13 – H14 | **111.31 °** |

| # | Angle | Value |
|---|---|---|
| 20 | H8 – C13 – H12 | **109.59 °** |
| 21 | H8 – C13 – H14 | **109.59 °** |
| 22 | H12 – C13 – H14 | **108.96 °** |

## Dihedral Angles

IUPAC signed convention, −180° to +180°.

| # | Dihedral | Value |
|---|---|---|
| 1 | H2 – C1 – N5 – C6 | **-64.88 °** |
| 2 | H2 – C1 – N5 – O7 | **-177.74 °** |
| 3 | H3 – C1 – N5 – C6 | **55.83 °** |
| 4 | H3 – C1 – N5 – O7 | **-57.03 °** |
| 5 | H4 – C1 – N5 – C6 | **176.74 °** |
| 6 | H4 – C1 – N5 – O7 | **63.88 °** |
| 7 | C1 – N5 – C6 – H9 | **-55.83 °** |
| 8 | C1 – N5 – C6 – H10 | **-176.74 °** |
| 9 | C1 – N5 – C6 – H11 | **64.88 °** |
| 10 | O7 – N5 – C6 – H9 | **57.03 °** |
| 11 | O7 – N5 – C6 – H10 | **-63.88 °** |
| 12 | O7 – N5 – C6 – H11 | **177.74 °** |
| 13 | C1 – N5 – O7 – C13 | **-121.68 °** |
| 14 | C6 – N5 – O7 – C13 | **121.68 °** |
| 15 | N5 – O7 – C13 – H8 | **-180.00 °** |
| 16 | N5 – O7 – C13 – H12 | **60.88 °** |
| 17 | N5 – O7 – C13 – H14 | **-60.88 °** |

# S2. N-Methoxy-N-methylaminomethyl

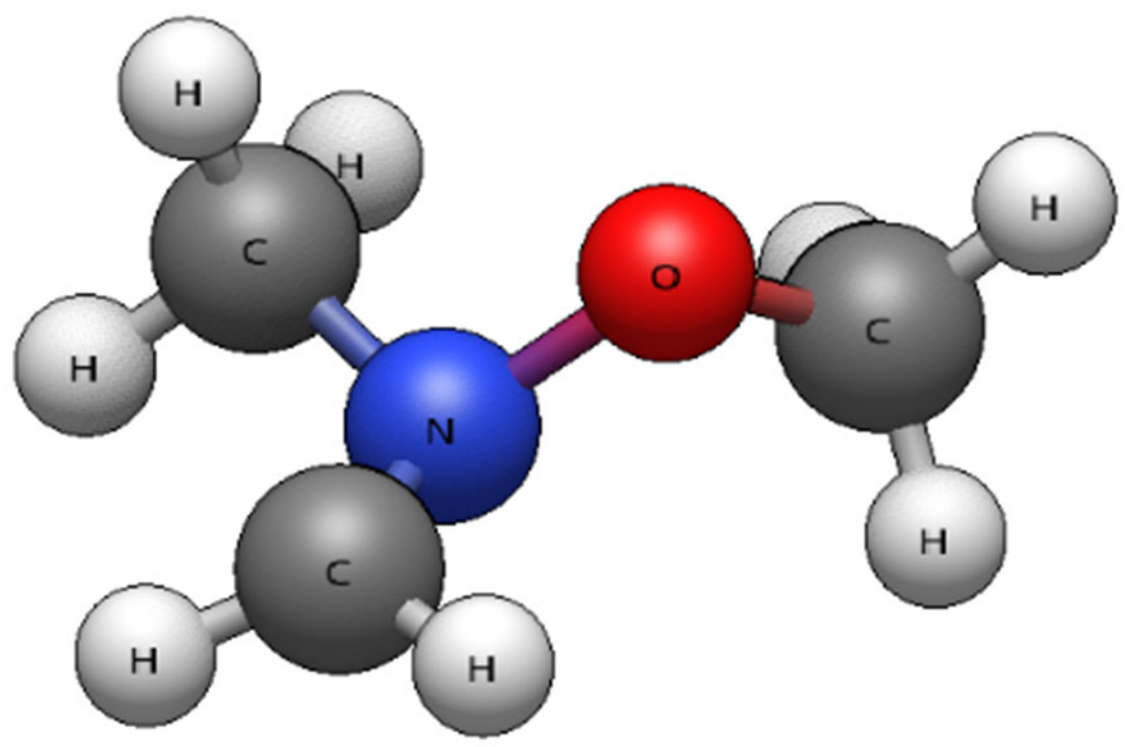


## Cartesian Coordinates

| # | Atom | X (Å) | Y (Å) | Z (Å) |
|---|---|---|---|---|
| 1 | C1 | 1.232801 | -1.186323 | 0.014859 |
| 2 | H2 | 2.241104 | -1.128290 | 0.399575 |
| 3 | H3 | 0.715634 | -2.132377 | -0.037957 |
| 4 | N4 | 0.440994 | -0.066954 | 0.244457 |
| 5 | C5 | 1.083991 | 1.223089 | 0.010306 |
| 6 | O6 | -0.742715 | -0.135879 | -0.561235 |
| 7 | H7 | -2.736605 | 0.040335 | -0.355154 |
| 8 | H8 | 1.350044 | 1.350581 | -1.043423 |
| 9 | H9 | 0.405830 | 2.019382 | 0.315591 |
| 10 | H10 | 1.982516 | 1.271084 | 0.626144 |
| 11 | H11 | -1.927425 | -0.707637 | 1.051792 |
| 12 | C12 | -1.859967 | 0.074312 | 0.291576 |
| 13 | H13 | -1.810332 | 1.051114 | 0.782692 |

## Bond Lengths

| # | Bond | Length |
|---|---|---|
| 1 | C1 – H2 | **1.0808 Å** |
| 2 | C1 – H3 | **1.0795 Å** |

| # | Bond | Length |
|---|---|---|
| 3 | C1 – N4 | **1.3902 Å** |
| 4 | N4 – C5 | **1.4603 Å** |
| 5 | N4 – O6 | **1.4335 Å** |
| 6 | C5 – H8 | **1.0942 Å** |
| 7 | C5 – H9 | **1.0896 Å** |
| 8 | C5 – H10 | **1.0904 Å** |
| 9 | O6 – C12 | **1.4212 Å** |
| 10 | H7 – C12 | **1.0899 Å** |
| 11 | H11 – C12 | **1.0927 Å** |
| 12 | C12 – H13 | **1.0944 Å** |

## Bond Angles

B is the vertex atom in each A–B–C triple.

| # | Angle | Value |
|---|---|---|
| 1 | H2 – C1 – H3 | **120.76 °** |
| 2 | H2 – C1 – N4 | **115.43 °** |
| 3 | H3 – C1 – N4 | **116.16 °** |
| 4 | C1 – N4 – C5 | **115.72 °** |
| 5 | C1 – N4 – O6 | **109.80 °** |
| 6 | C5 – N4 – O6 | **108.42 °** |
| 7 | N4 – C5 – H8 | **111.37 °** |
| 8 | N4 – C5 – H9 | **109.06 °** |
| 9 | N4 – C5 – H10 | **108.13 °** |
| 10 | H8 – C5 – H9 | **109.63 °** |
| 11 | H8 – C5 – H10 | **109.78 °** |
| 12 | H9 – C5 – H10 | **108.81 °** |
| 13 | N4 – O6 – C12 | **107.74 °** |
| 14 | O6 – C12 – H7 | **105.76 °** |
| 15 | O6 – C12 – H11 | **111.11 °** |
| 16 | O6 – C12 – H13 | **111.45 °** |
| 17 | H7 – C12 – H11 | **109.93 °** |
| 18 | H7 – C12 – H13 | **109.30 °** |
| 19 | H11 – C12 – H13 | **109.23 °** |

## Dihedral Angles

IUPAC signed convention, −180° to +180°.

| # | Dihedral | Value |
|---|---|---|
| 1 | H2 – C1 – N4 – C5 | **-43.77 °** |
| 2 | H2 – C1 – N4 – O6 | **-166.88 °** |
| 3 | H3 – C1 – N4 – C5 | **166.41 °** |
| 4 | H3 – C1 – N4 – O6 | **43.30 °** |
| 5 | C1 – N4 – C5 – H8 | **-64.45 °** |
| 6 | C1 – N4 – C5 – H9 | **174.42 °** |
| 7 | C1 – N4 – C5 – H10 | **56.25 °** |
| 8 | O6 – N4 – C5 – H8 | **59.38 °** |
| 9 | O6 – N4 – C5 – H9 | **-61.74 °** |
| 10 | O6 – N4 – C5 – H10 | **-179.92 °** |
| 11 | C1 – N4 – O6 – C12 | **-128.90 °** |
| 12 | C5 – N4 – O6 – C12 | **103.79 °** |
| 13 | N4 – O6 – C12 – H7 | **-178.62 °** |
| 14 | N4 – O6 – C12 – H11 | **62.12 °** |
| 15 | N4 – O6 – C12 – H13 | **-59.94 °** |

# S3. N,N-Dimethylaminoxymethyl radical

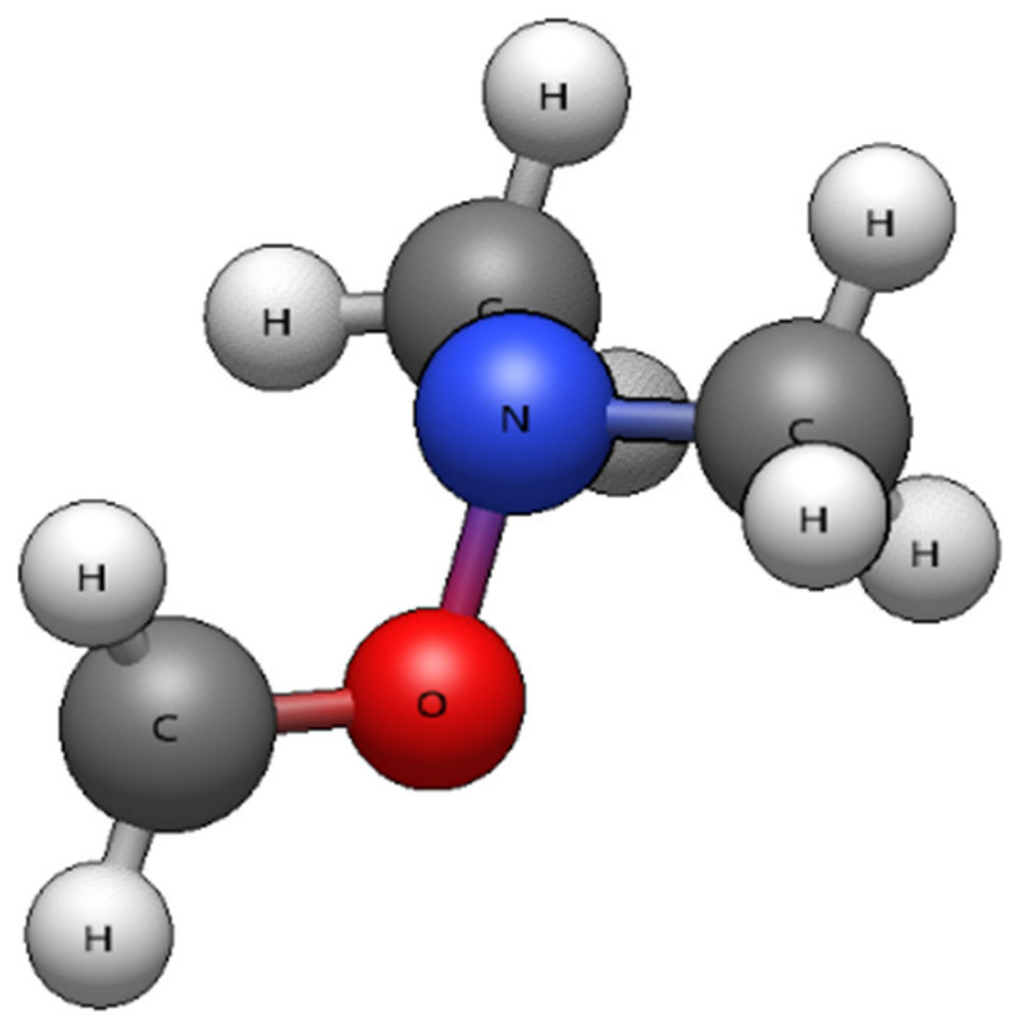


## Cartesian Coordinates

| # | Atom | X (Å) | Y (Å) | Z (Å) |
|---|---|---|---|---|
| 1 | C1 | 1.317656 | 1.071188 | -0.002244 |
| 2 | H2 | 2.242421 | 0.913363 | -0.558949 |
| 3 | H3 | 1.536480 | 1.071423 | 1.073321 |
| 4 | H4 | 0.890155 | 2.031395 | -0.285459 |
| 5 | N5 | 0.388585 | 0.007079 | -0.370115 |
| 6 | C6 | 0.903128 | -1.295309 | 0.054406 |
| 7 | O7 | -0.771856 | 0.254642 | 0.468350 |
| 8 | H8 | -2.802418 | 0.143583 | 0.345793 |
| 9 | H9 | 1.157961 | -1.307853 | 1.121725 |
| 10 | H10 | 0.147999 | -2.053307 | -0.148537 |
| 11 | H11 | 1.793563 | -1.513660 | -0.537399 |
| 12 | H12 | -1.861392 | 0.010599 | -1.298264 |
| 13 | C13 | -1.906173 | -0.064389 | -0.220366 |

### Bond Lengths

| # | Bond | Length |
|---|---|---|
| 1 | C1 – H2 | **1.0909 Å** |
| 2 | C1 – H3 | **1.0976 Å** |
| 3 | C1 – H4 | **1.0886 Å** |
| 4 | C1 – N5 | **1.4597 Å** |
| 5 | N5 – C6 | **1.4633 Å** |
| 6 | N5 – O7 | **1.4529 Å** |
| 7 | C6 – H9 | **1.0974 Å** |
| 8 | C6 – H10 | **1.0890 Å** |
| 9 | C6 – H11 | **1.0912 Å** |
| 10 | O7 – C13 | **1.3648 Å** |
| 11 | H8 – C13 | **1.0803 Å** |
| 12 | H12 – C13 | **1.0814 Å** |

## Bond Angles

B is the vertex atom in each A–B–C triple.

| # | Angle | Value |
|---|---|---|
| 1 | H2 – C1 – H3 | **109.34 °** |
| 2 | H2 – C1 – H4 | **109.13 °** |
| 3 | H2 – C1 – N5 | **107.79 °** |
| 4 | H3 – C1 – H4 | **109.45 °** |
| 5 | H3 – C1 – N5 | **111.96 °** |
| 6 | H4 – C1 – N5 | **109.12 °** |
| 7 | C1 – N5 – C6 | **110.60 °** |
| 8 | C1 – N5 – O7 | **103.81 °** |
| 9 | C6 – N5 – O7 | **105.37 °** |
| 10 | N5 – C6 – H9 | **111.96 °** |
| 11 | N5 – C6 – H10 | **108.76 °** |
| 12 | N5 – C6 – H11 | **107.92 °** |
| 13 | H9 – C6 – H10 | **109.53 °** |
| 14 | H9 – C6 – H11 | **109.61 °** |
| 15 | H10 – C6 – H11 | **108.99 °** |
| 16 | N5 – O7 – C13 | **109.44 °** |
| 17 | O7 – C13 – H8 | **112.34 °** |
| 18 | O7 – C13 – H12 | **116.89 °** |

| # | Angle | Value |
|---|---|---|
| 19 | H8 – C13 – H12 | **122.91 °** |

## Dihedral Angles

IUPAC signed convention, −180° to +180°.

| # | Dihedral | Value |
|---|---|---|
| 1 | H2 – C1 – N5 – C6 | **-64.32 °** |
| 2 | H2 – C1 – N5 – O7 | **-176.90 °** |
| 3 | H3 – C1 – N5 – C6 | **55.97 °** |
| 4 | H3 – C1 – N5 – O7 | **-56.61 °** |
| 5 | H4 – C1 – N5 – C6 | **177.29 °** |
| 6 | H4 – C1 – N5 – O7 | **64.71 °** |
| 7 | C1 – N5 – C6 – H9 | **-53.52 °** |
| 8 | C1 – N5 – C6 – H10 | **-174.69 °** |
| 9 | C1 – N5 – C6 – H11 | **67.20 °** |
| 10 | O7 – N5 – C6 – H9 | **58.07 °** |
| 11 | O7 – N5 – C6 – H10 | **-63.11 °** |
| 12 | O7 – N5 – C6 – H11 | **178.78 °** |
| 13 | C1 – N5 – O7 – C13 | **-144.67 °** |
| 14 | C6 – N5 – O7 – C13 | **99.01 °** |
| 15 | N5 – O7 – C13 – H8 | **177.47 °** |
| 16 | N5 – O7 – C13 – H12 | **27.35 °** |

# S4. Dimethyl Ether

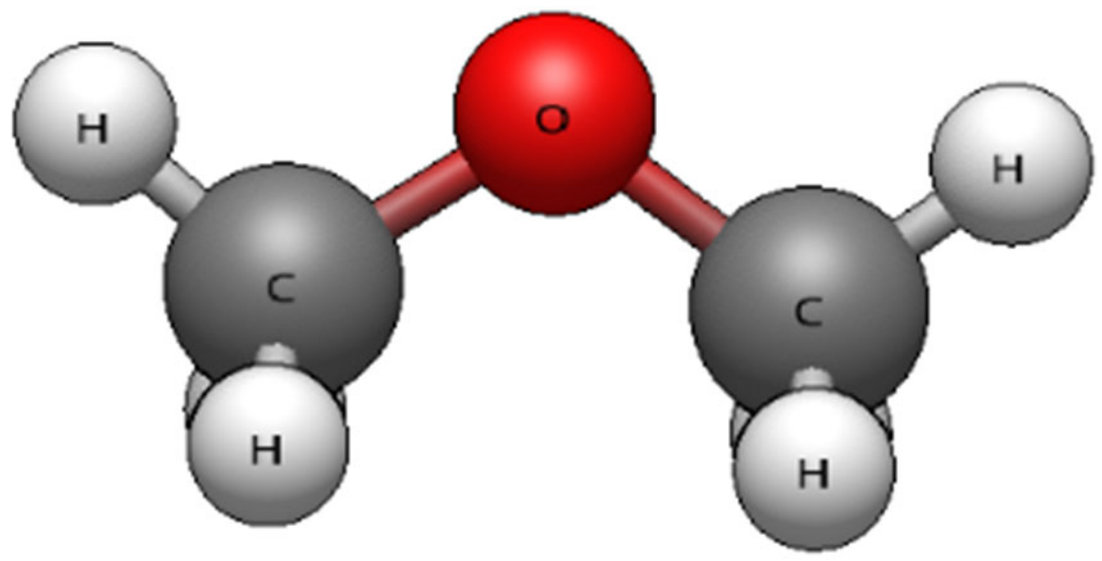


## Cartesian Coordinates

| # | Atom | X (Å) | Y (Å) | Z (Å) |
|---|---|---|---|---|
| 1 | H1 | -1.204620 | -0.891710 | -0.890154 |
| 2 | H2 | 1.204620 | -0.891710 | -0.890154 |
| 3 | H3 | 1.204620 | -0.891709 | 0.890155 |
| 4 | H4 | 2.021669 | 0.416414 | -0.000001 |
| 5 | H5 | -1.204620 | -0.891709 | 0.890155 |
| 6 | O6 | 0.000000 | 0.550951 | -0.000000 |
| 7 | C7 | -1.161058 | -0.250634 | -0.000000 |
| 8 | C8 | 1.161058 | -0.250634 | -0.000000 |
| 9 | H9 | -2.021669 | 0.416414 | -0.000001 |

## Bond Lengths

| # | Bond | Length |
|---|---|---|
| 1 | H1 – C7 | **1.0978 Å** |
| 2 | H2 – C8 | **1.0978 Å** |
| 3 | H3 – C8 | **1.0978 Å** |
| 4 | H4 – C8 | **1.0889 Å** |
| 5 | H5 – C7 | **1.0978 Å** |
| 6 | O6 – C7 | **1.4109 Å** |
| 7 | O6 – C8 | **1.4109 Å** |
| 8 | C7 – H9 | **1.0889 Å** |

## Bond Angles

B is the vertex atom in each A–B–C triple.

| # | Angle | Value |
|---|---|---|
| 1 | C7 – O6 – C8 | **110.76 °** |
| 2 | H1 – C7 – H5 | **108.35 °** |
| 3 | H1 – C7 – O6 | **111.37 °** |
| 4 | H1 – C7 – H9 | **109.05 °** |
| 5 | H5 – C7 – O6 | **111.37 °** |
| 6 | H5 – C7 – H9 | **109.05 °** |
| 7 | O6 – C7 – H9 | **107.60 °** |
| 8 | H2 – C8 – H3 | **108.35 °** |
| 9 | H2 – C8 – H4 | **109.05 °** |
| 10 | H2 – C8 – O6 | **111.37 °** |
| 11 | H3 – C8 – H4 | **109.05 °** |
| 12 | H3 – C8 – O6 | **111.37 °** |
| 13 | H4 – C8 – O6 | **107.60 °** |

## Dihedral Angles

IUPAC signed convention, −180° to +180°.

| # | Dihedral | Value |
|---|---|---|
| 1 | C8 – O6 – C7 – H1 | **60.54 °** |
| 2 | C8 – O6 – C7 – H5 | **-60.54 °** |
| 3 | C8 – O6 – C7 – H9 | **180.00 °** |
| 4 | C7 – O6 – C8 – H2 | **-60.54 °** |
| 5 | C7 – O6 – C8 – H3 | **60.54 °** |
| 6 | C7 – O6 – C8 – H4 | **-180.00 °** |

# S5. Dimethylaminomethoxymethyl radical

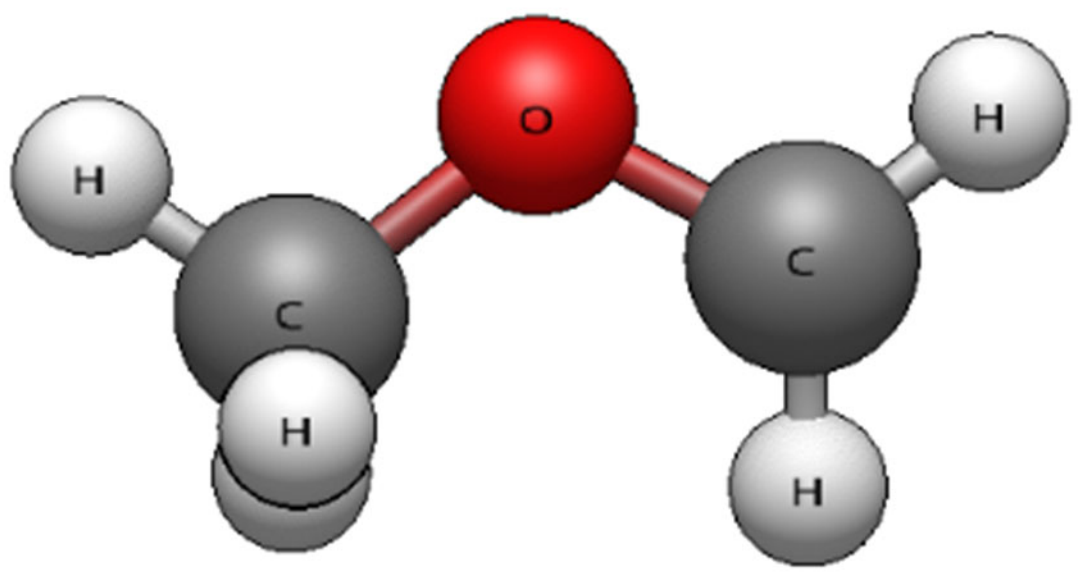


## Cartesian Coordinates

| # | Atom | X (Å) | Y (Å) | Z (Å) |
|---|---|---|---|---|
| 1 | H1 | -1.082480 | -1.299869 | 0.307259 |
| 2 | H2 | 1.250720 | -0.768834 | -0.955431 |
| 3 | H3 | 1.193544 | -0.928286 | 0.820737 |
| 4 | H4 | 1.961261 | 0.498154 | 0.078208 |
| 5 | H5 | -2.097362 | 0.266412 | 0.034194 |
| 6 | O6 | -0.065247 | 0.512712 | 0.013561 |
| 7 | C7 | -1.168591 | -0.277613 | -0.046875 |
| 8 | C8 | 1.151053 | -0.221597 | -0.014206 |

## Bond Lengths

| # | Bond | Length |
|---|---|---|
| 1 | H1 – C7 | **1.0853 Å** |
| 2 | H2 – C8 | **1.0933 Å** |
| 3 | H3 – C8 | **1.0947 Å** |
| 4 | H4 – C8 | **1.0877 Å** |
| 5 | H5 – C7 | **1.0794 Å** |
| 6 | O6 – C7 | **1.3585 Å** |
| 7 | O6 – C8 | **1.4210 Å** |

## Bond Angles

B is the vertex atom in each A–B–C triple.

| # | Angle | Value |
|---|---|---|
| 1 | C7 – O6 – C8 | **113.18 °** |
| 2 | H1 – C7 – H5 | **121.23 °** |
| 3 | H1 – C7 – O6 | **117.97 °** |
| 4 | H5 – C7 – O6 | **113.72 °** |
| 5 | H2 – C8 – H3 | **109.27 °** |
| 6 | H2 – C8 – H4 | **109.66 °** |
| 7 | H2 – C8 – O6 | **110.70 °** |
| 8 | H3 – C8 – H4 | **109.48 °** |
| 9 | H3 – C8 – O6 | **110.60 °** |
| 10 | H4 – C8 – O6 | **107.10 °** |

## Dihedral Angles

IUPAC signed convention, −180° to +180°.

| # | Dihedral | Value |
|---|---|---|
| 1 | C8 – O6 – C7 – H1 | **-25.48 °** |
| 2 | C8 – O6 – C7 – H5 | **-176.48 °** |
| 3 | C7 – O6 – C8 – H2 | **-62.68 °** |
| 4 | C7 – O6 – C8 – H3 | **58.57 °** |
| 5 | C7 – O6 – C8 – H4 | **177.81 °** |

# S6. Trimethylamine

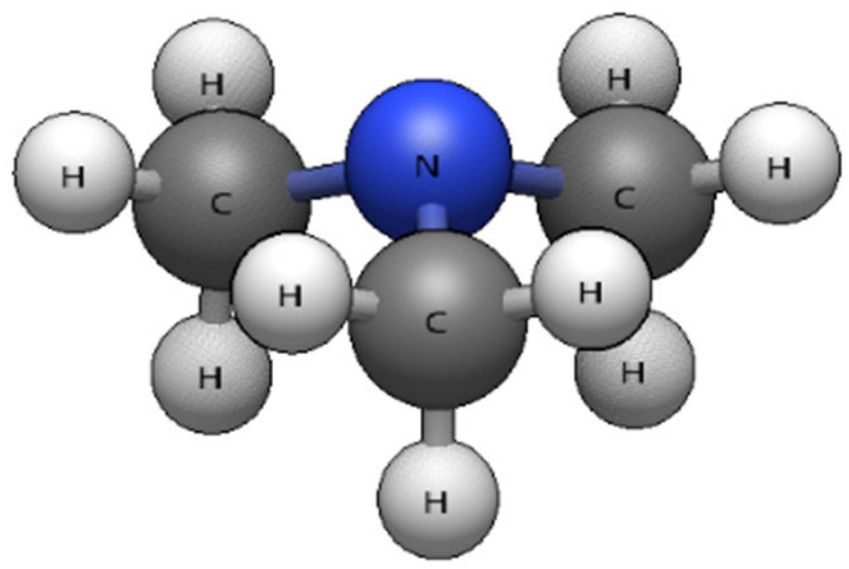


## Cartesian Coordinates

| # | Atom | X (Å) | Y (Å) | Z (Å) |
|---|---|---|---|---|
| 1 | N1 | 0.000000 | -0.000000 | -0.375211 |
| 2 | C2 | 0.351688 | 1.331748 | 0.097821 |
| 3 | C3 | 0.977483 | -0.970445 | 0.097821 |
| 4 | C4 | -1.329171 | -0.361303 | 0.097821 |
| 5 | H5 | 1.339875 | 1.606395 | -0.275784 |
| 6 | H6 | -0.372053 | 2.058481 | -0.275784 |
| 7 | H7 | 0.368418 | 1.395101 | 1.200773 |
| 8 | H8 | -2.061116 | 0.357168 | -0.275784 |
| 9 | H9 | -1.596671 | -1.351448 | -0.275784 |
| 10 | H10 | -1.392403 | -0.378490 | 1.200773 |
| 11 | H11 | 1.968724 | -0.707033 | -0.275784 |
| 12 | H12 | 1.023984 | -1.016611 | 1.200773 |
| 13 | H13 | 0.721241 | -1.963563 | -0.275784 |

## Bond Lengths

| # | Bond | Length |
|---|---|---|
| 1 | N1 – C2 | **1.4564 Å** |
| 2 | N1 – C3 | **1.4564 Å** |
| 3 | N1 – C4 | **1.4564 Å** |
| 4 | C2 – H5 | **1.0916 Å** |

| # | Bond | Length |
|---|---|---|
| 5 | C2 – H6 | **1.0916 Å** |
| 6 | C2 – H7 | **1.1049 Å** |
| 7 | C3 – H11 | **1.0916 Å** |
| 8 | C3 – H12 | **1.1049 Å** |
| 9 | C3 – H13 | **1.0916 Å** |
| 10 | C4 – H8 | **1.0916 Å** |
| 11 | C4 – H9 | **1.0916 Å** |
| 12 | C4 – H10 | **1.1049 Å** |

## Bond Angles

B is the vertex atom in each A–B–C triple.

| # | Angle | Value |
|---|---|---|
| 1 | C2 – N1 – C3 | **109.98 °** |
| 2 | C2 – N1 – C4 | **109.98 °** |
| 3 | C3 – N1 – C4 | **109.98 °** |
| 4 | N1 – C2 – H5 | **109.73 °** |
| 5 | N1 – C2 – H6 | **109.73 °** |
| 6 | N1 – C2 – H7 | **112.35 °** |
| 7 | H5 – C2 – H6 | **108.40 °** |
| 8 | H5 – C2 – H7 | **108.27 °** |
| 9 | H6 – C2 – H7 | **108.27 °** |
| 10 | N1 – C3 – H11 | **109.73 °** |
| 11 | N1 – C3 – H12 | **112.35 °** |
| 12 | N1 – C3 – H13 | **109.73 °** |
| 13 | H11 – C3 – H12 | **108.27 °** |
| 14 | H11 – C3 – H13 | **108.40 °** |
| 15 | H12 – C3 – H13 | **108.27 °** |
| 16 | N1 – C4 – H8 | **109.73 °** |
| 17 | N1 – C4 – H9 | **109.73 °** |
| 18 | N1 – C4 – H10 | **112.35 °** |
| 19 | H8 – C4 – H9 | **108.40 °** |
| 20 | H8 – C4 – H10 | **108.27 °** |
| 21 | H9 – C4 – H10 | **108.27 °** |

## Dihedral Angles

IUPAC signed convention, −180° to +180°.

| # | Dihedral | Value |
|---|---|---|
| 1 | C3 – N1 – C2 – H5 | **-59.86 °** |
| 2 | C3 – N1 – C2 – H6 | **-178.86 °** |
| 3 | C3 – N1 – C2 – H7 | **60.64 °** |
| 4 | C4 – N1 – C2 – H5 | **178.86 °** |
| 5 | C4 – N1 – C2 – H6 | **59.86 °** |
| 6 | C4 – N1 – C2 – H7 | **-60.64 °** |
| 7 | C2 – N1 – C3 – H11 | **59.86 °** |
| 8 | C2 – N1 – C3 – H12 | **-60.64 °** |
| 9 | C2 – N1 – C3 – H13 | **178.86 °** |
| 10 | C4 – N1 – C3 – H11 | **-178.86 °** |
| 11 | C4 – N1 – C3 – H12 | **60.64 °** |
| 12 | C4 – N1 – C3 – H13 | **-59.86 °** |
| 13 | C2 – N1 – C4 – H8 | **-59.86 °** |
| 14 | C2 – N1 – C4 – H9 | **-178.86 °** |
| 15 | C2 – N1 – C4 – H10 | **60.64 °** |
| 16 | C3 – N1 – C4 – H8 | **178.86 °** |
| 17 | C3 – N1 – C4 – H9 | **59.86 °** |
| 18 | C3 – N1 – C4 – H10 | **-60.64 °** |

# S7. Dimethylaminomethyl radical

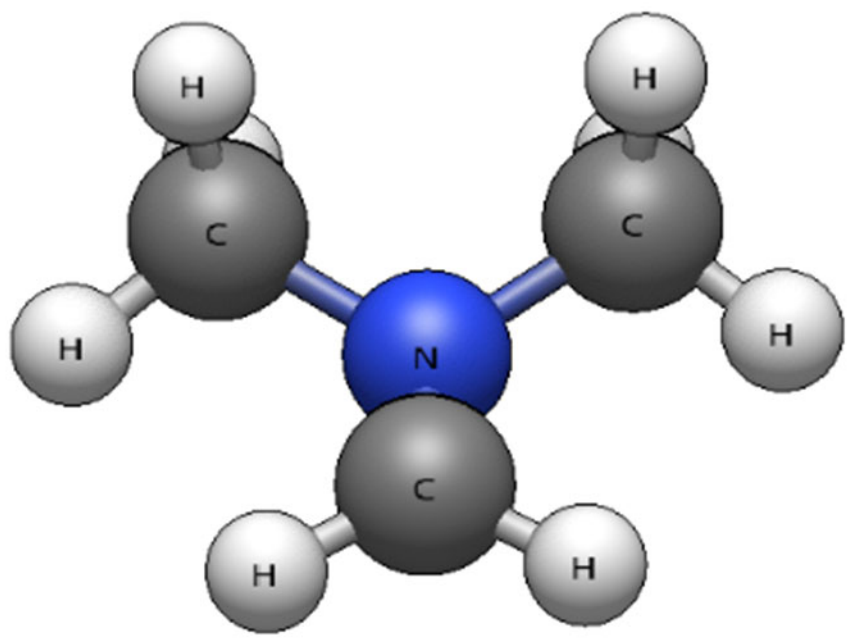


## Cartesian Coordinates

| # | Atom | X (Å) | Y (Å) | Z (Å) |
|---|---|---|---|---|
| 1 | N1 | 0.000114 | -0.056779 | -0.242691 |
| 2 | C2 | 1.216347 | 0.675583 | 0.064533 |
| 3 | C3 | -0.001464 | -1.399791 | 0.105831 |
| 4 | C4 | -1.213954 | 0.678800 | 0.066145 |
| 5 | H5 | 2.078928 | 0.101816 | -0.275574 |
| 6 | H6 | 1.207415 | 1.634367 | -0.456889 |
| 7 | H7 | 1.323629 | 0.861336 | 1.143182 |
| 8 | H8 | -1.202771 | 1.637889 | -0.454612 |
| 9 | H9 | -2.078421 | 0.107562 | -0.273542 |
| 10 | H10 | -1.319606 | 0.864047 | 1.145026 |
| 11 | H11 | 0.930331 | -1.932783 | -0.028088 |
| 12 | H12 | -0.934743 | -1.930367 | -0.027329 |

## Bond Lengths

| # | Bond | Length |
|---|---|---|
| 1 | N1 – C2 | **1.4526 Å** |
| 2 | N1 – C3 | **1.3875 Å** |
| 3 | N1 – C4 | **1.4527 Å** |
| 4 | C2 – H5 | **1.0904 Å** |
| 5 | C2 – H6 | **1.0914 Å** |

| # | Bond | Length |
|---|---|---|
| 6 | C2 – H7 | **1.0998 Å** |
| 7 | C3 – H11 | **1.0818 Å** |
| 8 | C3 – H12 | **1.0818 Å** |
| 9 | C4 – H8 | **1.0914 Å** |
| 10 | C4 – H9 | **1.0904 Å** |
| 11 | C4 – H10 | **1.0998 Å** |

## Bond Angles

B is the vertex atom in each A–B–C triple.

| # | Angle | Value |
|---|---|---|
| 1 | C2 – N1 – C3 | **115.84 °** |
| 2 | C2 – N1 – C4 | **113.55 °** |
| 3 | C3 – N1 – C4 | **115.83 °** |
| 4 | N1 – C2 – H5 | **109.34 °** |
| 5 | N1 – C2 – H6 | **109.57 °** |
| 6 | N1 – C2 – H7 | **111.98 °** |
| 7 | H5 – C2 – H6 | **108.65 °** |
| 8 | H5 – C2 – H7 | **108.52 °** |
| 9 | H6 – C2 – H7 | **108.72 °** |
| 10 | N1 – C3 – H11 | **116.41 °** |
| 11 | N1 – C3 – H12 | **116.41 °** |
| 12 | H11 – C3 – H12 | **119.09 °** |
| 13 | N1 – C4 – H8 | **109.57 °** |
| 14 | N1 – C4 – H9 | **109.33 °** |
| 15 | N1 – C4 – H10 | **111.97 °** |
| 16 | H8 – C4 – H9 | **108.65 °** |
| 17 | H8 – C4 – H10 | **108.73 °** |
| 18 | H9 – C4 – H10 | **108.52 °** |

## Dihedral Angles

IUPAC signed convention, −180° to +180°.

| # | Dihedral | Value |
|---|---|---|
| 1 | C3 – N1 – C2 – H5 | **-47.44 °** |

| # | Dihedral | Value |
|---|---|---|
| 2 | C3 – N1 – C2 – H6 | **-166.41 °** |
| 3 | C3 – N1 – C2 – H7 | **72.87 °** |
| 4 | C4 – N1 – C2 – H5 | **174.90 °** |
| 5 | C4 – N1 – C2 – H6 | **55.93 °** |
| 6 | C4 – N1 – C2 – H7 | **-64.79 °** |
| 7 | C2 – N1 – C3 – H11 | **37.39 °** |
| 8 | C2 – N1 – C3 – H12 | **-174.10 °** |
| 9 | C4 – N1 – C3 – H11 | **174.07 °** |
| 10 | C4 – N1 – C3 – H12 | **-37.41 °** |
| 11 | C2 – N1 – C4 – H8 | **-55.88 °** |
| 12 | C2 – N1 – C4 – H9 | **-174.86 °** |
| 13 | C2 – N1 – C4 – H10 | **64.84 °** |
| 14 | C3 – N1 – C4 – H8 | **166.46 °** |
| 15 | C3 – N1 – C4 – H9 | **47.48 °** |
| 16 | C3 – N1 – C4 – H10 | **-72.82 °** |

# S8. N,N,O-Trimethylhydroxylamine + Hydroxy radical

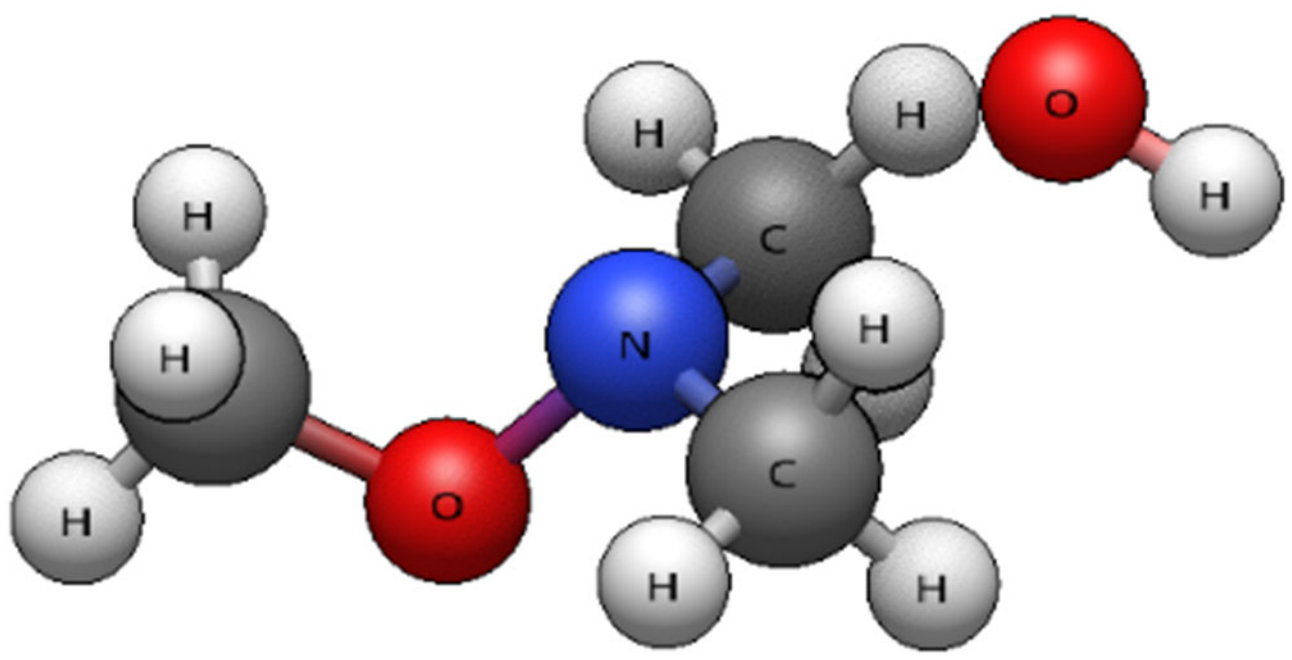


## Cartesian Coordinates

| # | Atom | X (Å) | Y (Å) | Z (Å) |
|---|---|---|---|---|
| 1 | C1 | 2.074366 | 1.510686 | 0.956886 |
| 2 | H2 | 2.658526 | 2.112795 | 0.186224 |
| 3 | H3 | 2.812417 | 1.085419 | 1.652513 |
| 4 | H4 | 1.407424 | 2.189719 | 1.484158 |
| 5 | N5 | 1.307731 | 0.545457 | 0.235191 |
| 6 | C6 | 2.048806 | -0.613407 | -0.228036 |
| 7 | O7 | 0.202856 | 0.123478 | 1.038836 |
| 8 | H8 | -1.802761 | -0.007782 | 0.985287 |
| 9 | H9 | 2.461482 | -1.207630 | 0.599844 |
| 10 | H10 | 1.398952 | -1.248642 | -0.829806 |
| 11 | H11 | 2.863491 | -0.253061 | -0.857231 |
| 12 | H12 | -1.149552 | 1.395261 | 0.091185 |
| 13 | C13 | -1.007426 | 0.336100 | 0.324087 |
| 14 | H14 | -1.025009 | -0.240141 | -0.605794 |

| | | | | |
|---|---|---|---|---|
| 15 | O15 | 3.767312 | 2.404287 | -0.955583 |
| 16 | H16 | 4.547167 | 2.243163 | -0.395573 |

## Bond Lengths

| # | Bond | Length |
|---|---|---|
| 1 | C1 – H2 | **1.1392 Å** |
| 2 | C1 – H3 | **1.0998 Å** |
| 3 | C1 – H4 | **1.0881 Å** |
| 4 | C1 – N5 | **1.4284 Å** |
| 5 | N5 – C6 | **1.4515 Å** |
| 6 | N5 – O7 | **1.4299 Å** |
| 7 | C6 – H9 | **1.0994 Å** |
| 8 | C6 – H10 | **1.0899 Å** |
| 9 | C6 – H11 | **1.0906 Å** |
| 10 | O7 – C13 | **1.4216 Å** |
| 11 | H8 – C13 | **1.0900 Å** |
| 12 | H12 – C13 | **1.0937 Å** |
| 13 | C13 – H14 | **1.0941 Å** |
| 14 | O15 – H16 | **0.9735 Å** |

## Bond Angles

B is the vertex atom in each A–B–C triple.

| # | Angle | Value |
|---|---|---|
| 1 | H2 – C1 – H3 | **106.75 °** |
| 2 | H2 – C1 – H4 | **108.20 °** |
| 3 | H2 – C1 – N5 | **106.89 °** |
| 4 | H3 – C1 – H4 | **110.25 °** |
| 5 | H3 – C1 – N5 | **114.74 °** |

| | | |
|---|---|---|
| 6 | H4 – C1 – N5 | **109.73 °** |
| 7 | C1 – N5 – C6 | **115.26 °** |
| 8 | C1 – N5 – O7 | **109.28 °** |
| 9 | C6 – N5 – O7 | **109.77 °** |
| 10 | N5 – C6 – H9 | **112.51 °** |
| 11 | N5 – C6 – H10 | **109.70 °** |
| 12 | N5 – C6 – H11 | **107.56 °** |
| 13 | H9 – C6 – H10 | **108.94 °** |
| 14 | H9 – C6 – H11 | **109.43 °** |
| 15 | H10 – C6 – H11 | **108.63 °** |
| 16 | N5 – O7 – C13 | **109.34 °** |
| 17 | O7 – C13 – H8 | **105.61 °** |
| 18 | O7 – C13 – H12 | **111.26 °** |
| 19 | O7 – C13 – H14 | **111.24 °** |
| 20 | H8 – C13 – H12 | **109.87 °** |
| 21 | H8 – C13 – H14 | **109.74 °** |
| 22 | H12 – C13 – H14 | **109.08 °** |

## Dihedral Angles

IUPAC signed convention, −180° to +180°.

| # | Dihedral | Value |
|---|---|---|
| 1 | H2 – C1 – N5 – C6 | **-78.01 °** |
| 2 | H2 – C1 – N5 – O7 | **157.84 °** |
| 3 | H3 – C1 – N5 – C6 | **40.12 °** |
| 4 | H3 – C1 – N5 – O7 | **-84.03 °** |
| 5 | H4 – C1 – N5 – C6 | **164.88 °** |
| 6 | H4 – C1 – N5 – O7 | **40.73 °** |
| 7 | C1 – N5 – C6 – H9 | **-62.94 °** |

| | | |
|---|---|---|
| 8 | C1 – N5 – C6 – H10 | **175.62 °** |
| 9 | C1 – N5 – C6 – H11 | **57.64 °** |
| 10 | O7 – N5 – C6 – H9 | **60.95 °** |
| 11 | O7 – N5 – C6 – H10 | **-60.49 °** |
| 12 | O7 – N5 – C6 – H11 | **-178.47 °** |
| 13 | C1 – N5 – O7 – C13 | **-124.20 °** |
| 14 | C6 – N5 – O7 – C13 | **108.49 °** |
| 15 | N5 – O7 – C13 – H8 | **-179.74 °** |
| 16 | N5 – O7 – C13 – H12 | **61.09 °** |
| 17 | N5 – O7 – C13 – H14 | **-60.75 °** |

# S9. N,N,O-Trimethylhydroxylamine + Hydroxy radical TS

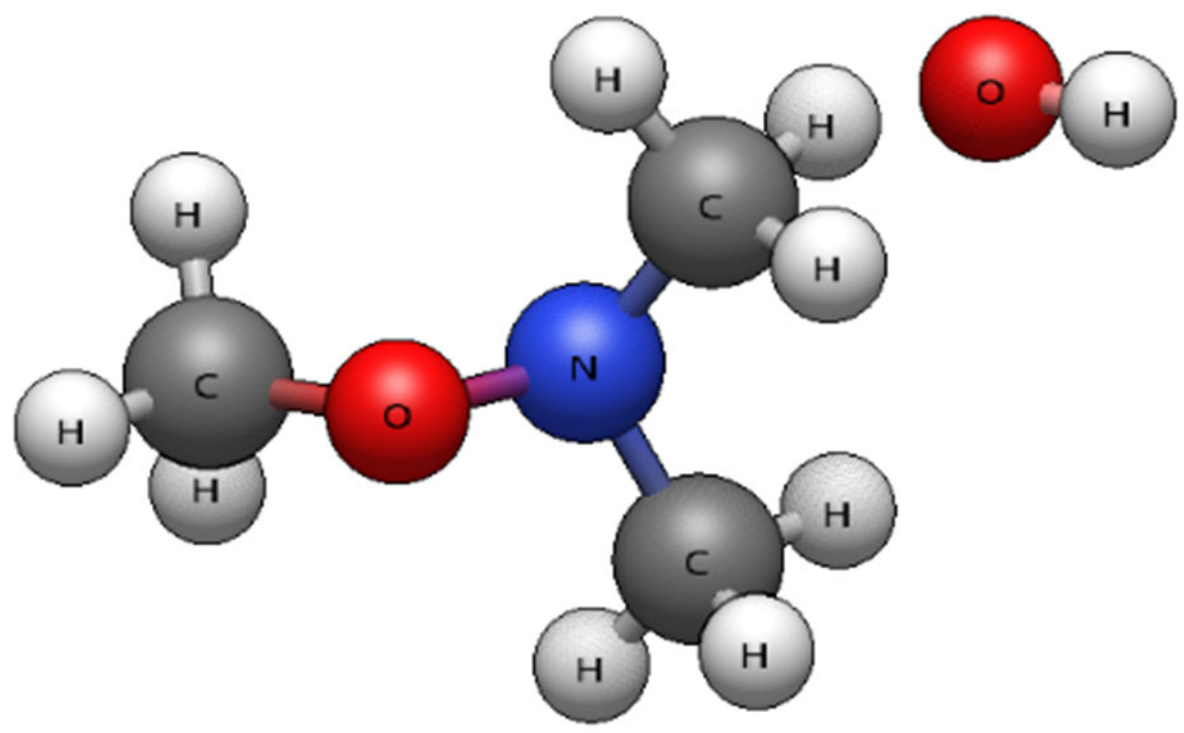


## Cartesian Coordinates

| # | Atom | X (Å) | Y (Å) | Z (Å) |
|---|---|---|---|---|
| 1 | C1 | 2.109775 | 1.514292 | 0.913621 |
| 2 | H2 | 2.733923 | 2.082803 | 0.072964 |
| 3 | H3 | 2.870105 | 1.106502 | 1.618307 |
| 4 | H4 | 1.461261 | 2.246995 | 1.417208 |
| 5 | N5 | 1.325509 | 0.540442 | 0.243307 |
| 6 | C6 | 2.047665 | -0.620747 | -0.232374 |
| 7 | O7 | 0.213135 | 0.152045 | 1.034427 |
| 8 | H8 | -1.794763 | -0.010747 | 0.991499 |
| 9 | H9 | 2.463470 | -1.230904 | 0.600135 |
| 10 | H10 | 1.382927 | -1.254654 | -0.843279 |
| 11 | H11 | 2.871295 | -0.251219 | -0.866676 |
| 12 | H12 | -1.159482 | 1.410306 | 0.076883 |
| 13 | C13 | -0.993042 | 0.343030 | 0.320962 |
| 14 | H14 | -1.015299 | -0.246861 | -0.616915 |
| 15 | O15 | 3.723379 | 2.385611 | -0.931668 |
| 16 | H16 | 4.534554 | 2.250943 | -0.395140 |

## Bond Lengths

| # | Bond | Length |
|---|---|---|
| 1 | C1 – H2 | **1.1914 Å** |
| 2 | C1 – H3 | **1.1140 Å** |
| 3 | C1 – H4 | **1.1005 Å** |
| 4 | C1 – N5 | **1.4187 Å** |
| 5 | N5 – C6 | **1.4478 Å** |
| 6 | N5 – O7 | **1.4192 Å** |
| 7 | C6 – H9 | **1.1128 Å** |
| 8 | C6 – H10 | **1.1031 Å** |
| 9 | C6 – H11 | **1.1033 Å** |
| 10 | O7 – C13 | **1.4143 Å** |
| 11 | H8 – C13 | **1.1034 Å** |
| 12 | H12 – C13 | **1.1074 Å** |
| 13 | C13 – H14 | **1.1082 Å** |
| 14 | O15 – H16 | **0.9818 Å** |

## Bond Angles

B is the vertex atom in each A–B–C triple.

| # | Angle | Value |
|---|---|---|
| 1 | H2 – C1 – H3 | **105.28 °** |
| 2 | H2 – C1 – H4 | **108.29 °** |
| 3 | H2 – C1 – N5 | **106.48 °** |
| 4 | H3 – C1 – H4 | **110.88 °** |
| 5 | H3 – C1 – N5 | **115.14 °** |
| 6 | H4 – C1 – N5 | **110.33 °** |
| 7 | C1 – N5 – C6 | **115.47 °** |
| 8 | C1 – N5 – O7 | **110.96 °** |
| 9 | C6 – N5 – O7 | **110.77 °** |
| 10 | N5 – C6 – H9 | **112.36 °** |
| 11 | N5 – C6 – H10 | **110.01 °** |
| 12 | N5 – C6 – H11 | **107.02 °** |
| 13 | H9 – C6 – H10 | **108.93 °** |
| 14 | H9 – C6 – H11 | **109.56 °** |
| 15 | H10 – C6 – H11 | **108.90 °** |
| 16 | N5 – O7 – C13 | **110.50 °** |

| # | Angle | Value |
|---|---|---|
| 17 | O7 – C13 – H8 | **105.65 °** |
| 18 | O7 – C13 – H12 | **111.68 °** |
| 19 | O7 – C13 – H14 | **111.85 °** |
| 20 | H8 – C13 – H12 | **109.50 °** |
| 21 | H8 – C13 – H14 | **109.21 °** |
| 22 | H12 – C13 – H14 | **108.87 °** |

## Dihedral Angles

IUPAC signed convention, −180° to +180°.

| # | Dihedral | Value |
|---|---|---|
| 1 | H2 – C1 – N5 – C6 | **-74.42 °** |
| 2 | H2 – C1 – N5 – O7 | **158.48 °** |
| 3 | H3 – C1 – N5 – C6 | **41.83 °** |
| 4 | H3 – C1 – N5 – O7 | **-85.26 °** |
| 5 | H4 – C1 – N5 – C6 | **168.27 °** |
| 6 | H4 – C1 – N5 – O7 | **41.17 °** |
| 7 | C1 – N5 – C6 – H9 | **-65.43 °** |
| 8 | C1 – N5 – C6 – H10 | **173.03 °** |
| 9 | C1 – N5 – C6 – H11 | **54.87 °** |
| 10 | O7 – N5 – C6 – H9 | **61.76 °** |
| 11 | O7 – N5 – C6 – H10 | **-59.78 °** |
| 12 | O7 – N5 – C6 – H11 | **-177.94 °** |
| 13 | C1 – N5 – O7 – C13 | **-124.21 °** |
| 14 | C6 – N5 – O7 – C13 | **106.16 °** |
| 15 | N5 – O7 – C13 – H8 | **-178.26 °** |
| 16 | N5 – O7 – C13 – H12 | **62.77 °** |
| 17 | N5 – O7 – C13 – H14 | **-59.54 °** |

# S10. N-Methoxy-N-methylaminomethyl radical + water

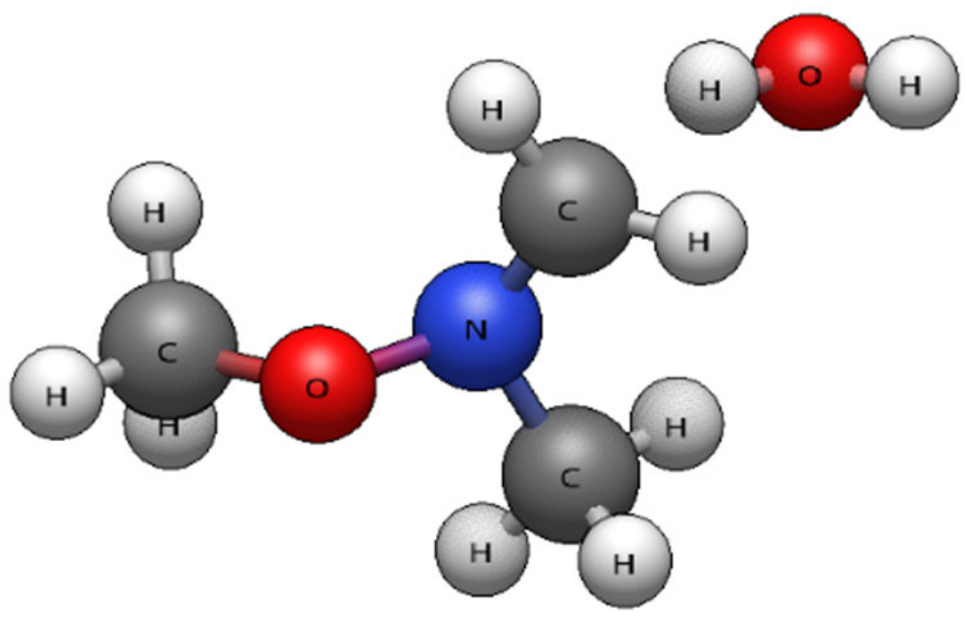

## Cartesian Coordinates

| # | Atom | X (Å) | Y (Å) | Z (Å) |
|---|---|---|---|---|
| 1 | C1 | 2.009246 | 1.400868 | 1.147737 |
| 2 | H2 | 3.101294 | 2.314147 | -0.453314 |
| 3 | H3 | 3.015824 | 1.167595 | 1.479457 |
| 4 | H4 | 1.438522 | 2.169410 | 1.644583 |
| 5 | N5 | 1.313156 | 0.497199 | 0.405484 |
| 6 | C6 | 2.025610 | -0.635083 | -0.165280 |
| 7 | O7 | 0.097427 | 0.053057 | 1.071011 |
| 8 | H8 | -1.893520 | -0.011599 | 0.871366 |
| 9 | H9 | 2.438303 | -1.290305 | 0.610258 |
| 10 | H10 | 1.346785 | -1.209839 | -0.792956 |
| 11 | H11 | 2.831494 | -0.247077 | -0.788566 |
| 12 | H12 | -1.143947 | 1.388770 | 0.060295 |
| 13 | C13 | -1.046000 | 0.320745 | 0.271322 |
| 14 | H14 | -1.028584 | -0.236768 | -0.670424 |
| 15 | O15 | 3.850127 | 2.429107 | -1.064385 |
| 16 | H16 | 4.627305 | 2.349743 | -0.502253 |

## Bond Lengths

| # | Bond | Length |
|---|---|---|
| 1 | C1 – H3 | **1.0852 Å** |
| 2 | C1 – H4 | **1.0785 Å** |
| 3 | C1 – N5 | **1.3609 Å** |
| 4 | H2 – O15 | **0.9733 Å** |
| 5 | N5 – C6 | **1.4544 Å** |
| 6 | N5 – O7 | **1.4554 Å** |
| 7 | C6 – H9 | **1.0959 Å** |
| 8 | C6 – H10 | **1.0886 Å** |
| 9 | C6 – H11 | **1.0902 Å** |
| 10 | O7 – C13 | **1.4208 Å** |
| 11 | H8 – C13 | **1.0903 Å** |
| 12 | H12 – C13 | **1.0931 Å** |
| 13 | C13 – H14 | **1.0945 Å** |
| 14 | O15 – H16 | **0.9624 Å** |

## Bond Angles

B is the vertex atom in each A–B–C triple.

| # | Angle | Value |
|---|---|---|
| 1 | H3 – C1 – H4 | **120.21 °** |
| 2 | H3 – C1 – N5 | **119.89 °** |
| 3 | H4 – C1 – N5 | **116.98 °** |
| 4 | C1 – N5 – C6 | **118.71 °** |
| 5 | C1 – N5 – O7 | **112.36 °** |
| 6 | C6 – N5 – O7 | **110.55 °** |
| 7 | N5 – C6 – H9 | **111.85 °** |

| | | |
|---|---|---|
| 8 | N5 – C6 – H10 | **109.38 °** |
| 9 | N5 – C6 – H11 | **108.02 °** |
| 10 | H9 – C6 – H10 | **109.10 °** |
| 11 | H9 – C6 – H11 | **109.82 °** |
| 12 | H10 – C6 – H11 | **108.62 °** |
| 13 | N5 – O7 – C13 | **110.94 °** |
| 14 | O7 – C13 – H8 | **104.97 °** |
| 15 | O7 – C13 – H12 | **111.40 °** |
| 16 | O7 – C13 – H14 | **112.06 °** |
| 17 | H8 – C13 – H12 | **109.54 °** |
| 18 | H8 – C13 – H14 | **109.31 °** |
| 19 | H12 – C13 – H14 | **109.45 °** |
| 20 | H2 – O15 – H16 | **104.17 °** |

## Dihedral Angles

IUPAC signed convention, −180° to +180°.

| # | Dihedral | Value |
|---|---|---|
| 1 | H3 – C1 – N5 – C6 | **12.89 °** |
| 2 | H3 – C1 – N5 – O7 | **-118.28 °** |
| 3 | H4 – C1 – N5 – C6 | **173.63 °** |
| 4 | H4 – C1 – N5 – O7 | **42.47 °** |
| 5 | C1 – N5 – C6 – H9 | **-63.72 °** |
| 6 | C1 – N5 – C6 – H10 | **175.30 °** |
| 7 | C1 – N5 – C6 – H11 | **57.24 °** |
| 8 | O7 – N5 – C6 – H9 | **68.24 °** |
| 9 | O7 – N5 – C6 – H10 | **-52.74 °** |
| 10 | O7 – N5 – C6 – H11 | **-170.79 °** |
| 11 | C1 – N5 – O7 – C13 | **-121.99 °** |

| | | |
|---|---|---|
| 12 | C6 – N5 – O7 – C13 | **102.85 °** |
| 13 | N5 – O7 – C13 – H8 | **177.91 °** |
| 14 | N5 – O7 – C13 – H12 | **59.45 °** |
| 15 | N5 – O7 – C13 – H14 | **-63.55 °** |

# S11. N-Methoxy-N-methylaminomethyl radical fragmentation TS

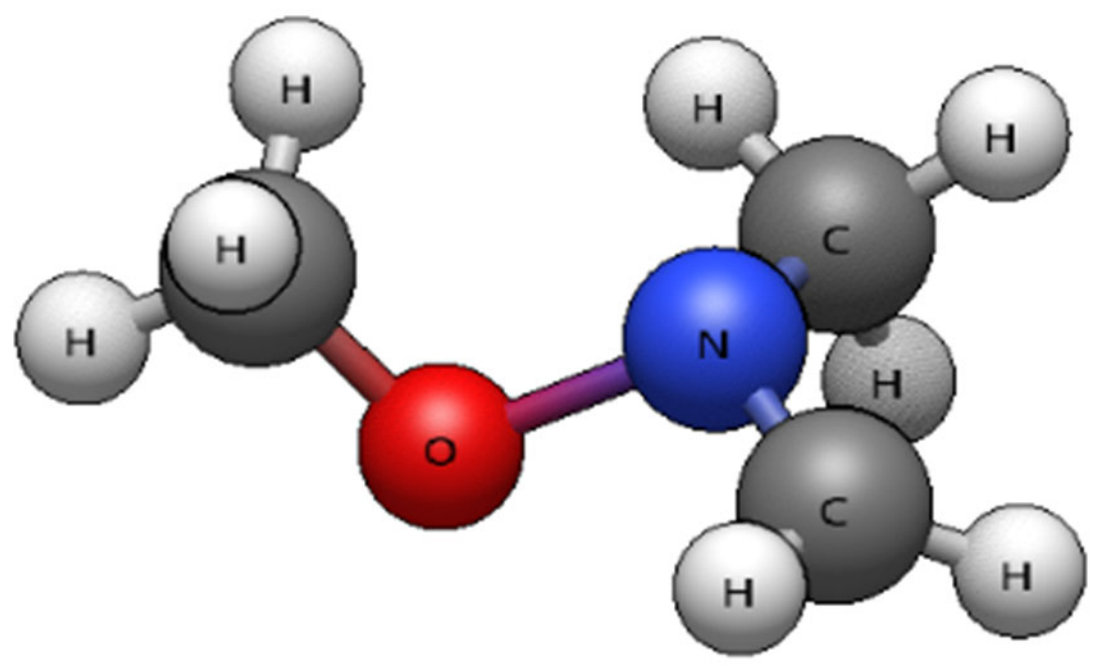


## Cartesian Coordinates

| # | Atom | X (Å) | Y (Å) | Z (Å) |
|---|---|---|---|---|
| 1 | C1 | 1.285363 | -1.176965 | 0.136414 |
| 2 | H2 | 2.307541 | -1.097525 | -0.225151 |
| 3 | H3 | 0.840276 | -2.151065 | 0.282707 |
| 4 | N4 | 0.540556 | -0.111442 | 0.318110 |
| 5 | C5 | 1.113212 | 1.201768 | 0.066872 |
| 6 | O6 | -0.853433 | -0.167820 | -0.592241 |
| 7 | H7 | -2.826021 | 0.130053 | -0.383878 |
| 8 | H8 | 1.477483 | 1.296898 | -0.959964 |
| 9 | H9 | 0.357593 | 1.964129 | 0.245535 |
| 10 | H10 | 1.938475 | 1.359852 | 0.762961 |
| 11 | H11 | -2.079423 | -0.669457 | 1.018929 |
| 12 | C12 | -1.939412 | 0.105389 | 0.256894 |
| 13 | H13 | -1.848928 | 1.078897 | 0.756346 |

## Bond Lengths

| # | Bond | Length |
|---|---|---|
| 1 | C1 – H2 | **1.0871 Å** |
| 2 | C1 – H3 | **1.0809 Å** |

| # | Bond | Length |
|---|---|---|
| 3 | C1 – N4 | **1.3127 Å** |
| 4 | N4 – C5 | **1.4545 Å** |
| 5 | N4 – O6 | **1.6659 Å** |
| 6 | C5 – H8 | **1.0937 Å** |
| 7 | C5 – H9 | **1.0882 Å** |
| 8 | C5 – H10 | **1.0911 Å** |
| 9 | O6 – C12 | **1.4054 Å** |
| 10 | H7 – C12 | **1.0942 Å** |
| 11 | H11 – C12 | **1.0958 Å** |
| 12 | C12 – H13 | **1.0979 Å** |

## Bond Angles

B is the vertex atom in each A–B–C triple.

| # | Angle | Value |
|---|---|---|
| 1 | H2 – C1 – H3 | **119.87 °** |
| 2 | H2 – C1 – N4 | **121.35 °** |
| 3 | H3 – C1 – N4 | **118.63 °** |
| 4 | C1 – N4 – C5 | **119.05 °** |
| 5 | C1 – N4 – O6 | **111.82 °** |
| 6 | C5 – N4 – O6 | **105.40 °** |
| 7 | N4 – C5 – H8 | **111.83 °** |
| 8 | N4 – C5 – H9 | **109.32 °** |
| 9 | N4 – C5 – H10 | **108.57 °** |
| 10 | H8 – C5 – H9 | **108.94 °** |
| 11 | H8 – C5 – H10 | **109.54 °** |
| 12 | H9 – C5 – H10 | **108.60 °** |
| 13 | N4 – O6 – C12 | **108.05 °** |
| 14 | O6 – C12 – H7 | **106.06 °** |
| 15 | O6 – C12 – H11 | **112.42 °** |
| 16 | O6 – C12 – H13 | **112.55 °** |
| 17 | H7 – C12 – H11 | **108.64 °** |
| 18 | H7 – C12 – H13 | **108.25 °** |
| 19 | H11 – C12 – H13 | **108.73 °** |

## Dihedral Angles

IUPAC signed convention, −180° to +180°.

| # | Dihedral | Value |
|---|---|---|
| 1 | H2 – C1 – N4 – C5 | **1.85 °** |
| 2 | H2 – C1 – N4 – O6 | **-121.49 °** |
| 3 | H3 – C1 – N4 – C5 | **177.32 °** |
| 4 | H3 – C1 – N4 – O6 | **53.98 °** |
| 5 | C1 – N4 – C5 – H8 | **-58.10 °** |
| 6 | C1 – N4 – C5 – H9 | **-178.81 °** |
| 7 | C1 – N4 – C5 – H10 | **62.87 °** |
| 8 | O6 – N4 – C5 – H8 | **68.35 °** |
| 9 | O6 – N4 – C5 – H9 | **-52.37 °** |
| 10 | O6 – N4 – C5 – H10 | **-170.68 °** |
| 11 | C1 – N4 – O6 – C12 | **-129.78 °** |
| 12 | C5 – N4 – O6 – C12 | **99.47 °** |
| 13 | N4 – O6 – C12 – H7 | **-176.11 °** |
| 14 | N4 – O6 – C12 – H11 | **65.29 °** |
| 15 | N4 – O6 – C12 – H13 | **-57.91 °** |

# S12. N-methylmethanimine

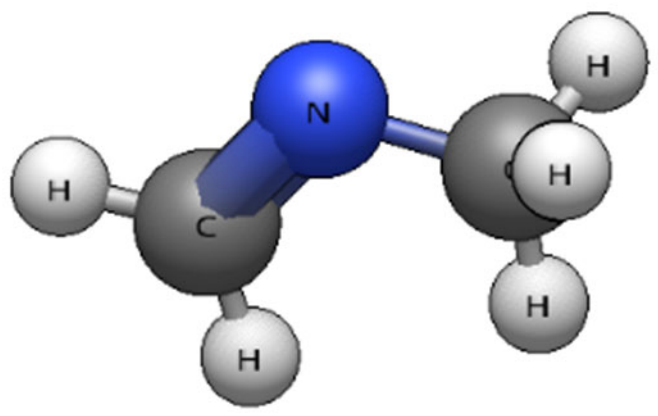


## Cartesian Coordinates

| # | Atom | X (Å) | Y (Å) | Z (Å) |
|---|---|---|---|---|
| 1 | C1 | 1.136291 | -1.134075 | 0.192344 |
| 2 | H2 | 2.126111 | -1.057560 | 0.658217 |
| 3 | H3 | 0.698912 | -2.124029 | 0.093819 |
| 4 | N4 | 0.487246 | -0.124735 | -0.232172 |
| 5 | C5 | 1.134157 | 1.171534 | -0.067635 |
| 6 | H6 | 2.119821 | 1.105558 | 0.411817 |
| 7 | H7 | 1.241824 | 1.638518 | -1.048110 |
| 8 | H8 | 0.486228 | 1.815379 | 0.529565 |

## Bond Lengths

| # | Bond | Length |
|---|---|---|
| 1 | C1 – H2 | **1.0966 Å** |
| 2 | C1 – H3 | **1.0867 Å** |
| 3 | C1 – N4 | **1.2729 Å** |
| 4 | N4 – C5 | **1.4580 Å** |
| 5 | C5 – H6 | **1.0981 Å** |
| 6 | C5 – H7 | **1.0913 Å** |
| 7 | C5 – H8 | **1.0913 Å** |

## Bond Angles

B is the vertex atom in each A–B–C triple.

| # | Angle | Value |
|---|---|---|
| 1 | H2 – C1 – H3 | **117.73 °** |
| 2 | H2 – C1 – N4 | **123.13 °** |
| 3 | H3 – C1 – N4 | **119.14 °** |
| 4 | C1 – N4 – C5 | **116.17 °** |
| 5 | N4 – C5 – H6 | **113.21 °** |
| 6 | N4 – C5 – H7 | **108.83 °** |
| 7 | N4 – C5 – H8 | **108.83 °** |
| 8 | H6 – C5 – H7 | **109.23 °** |
| 9 | H6 – C5 – H8 | **109.23 °** |
| 10 | H7 – C5 – H8 | **107.32 °** |

## Dihedral Angles

IUPAC signed convention, −180° to +180°.

| # | Dihedral | Value |
|---|---|---|
| 1 | H2 – C1 – N4 – C5 | **0.00 °** |
| 2 | H3 – C1 – N4 – C5 | **180.00 °** |
| 3 | C1 – N4 – C5 – H6 | **-0.02 °** |
| 4 | C1 – N4 – C5 – H7 | **-121.69 °** |
| 5 | C1 – N4 – C5 – H8 | **121.64 °** |

# S13. Methoxy radical

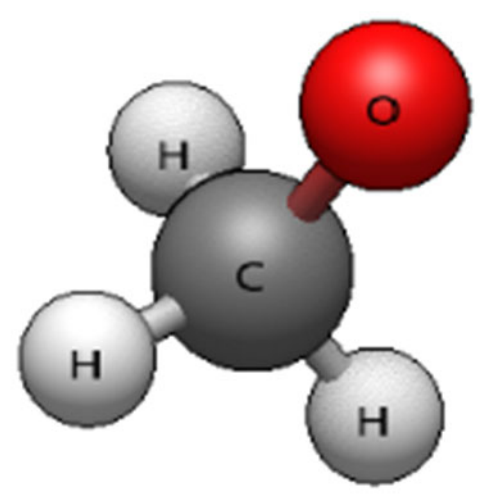


## Cartesian Coordinates

| # | Atom | X (Å) | Y (Å) | Z (Å) |
|---|---|---|---|---|
| 1 | C1 | -1.830359 | 0.039428 | 0.302893 |
| 2 | H2 | -1.823372 | 1.004969 | 0.820816 |
| 3 | H3 | -2.728871 | 0.024198 | -0.337421 |
| 4 | H4 | -1.928006 | -0.791581 | 1.010332 |
| 5 | O5 | -0.769415 | -0.113409 | -0.559839 |

## Bond Lengths

| # | Bond | Length |
|---|---|---|
| 1 | C1 – H2 | **1.0957 Å** |
| 2 | C1 – H3 | **1.1034 Å** |
| 3 | C1 – H4 | **1.0957 Å** |
| 4 | C1 – O5 | **1.3760 Å** |

## Bond Angles

B is the vertex atom in each A–B–C triple.

| # | Angle | Value |
|---|---|---|
| 1 | H2 – C1 – H3 | **106.96 °** |
| 2 | H2 – C1 – H4 | **111.33 °** |
| 3 | H2 – C1 – O5 | **112.91 °** |
| 4 | H3 – C1 – H4 | **106.96 °** |

| # | Angle | Value |
|---|---|---|
| 5 | H3 – C1 – O5 | **105.22 °** |
| 6 | H4 – C1 – O5 | **112.91 °** |

## Dihedral Angles

No dihedral angles found.

# S14. N,N,O-Trimethylhydroxylamine + Hydroxy radical

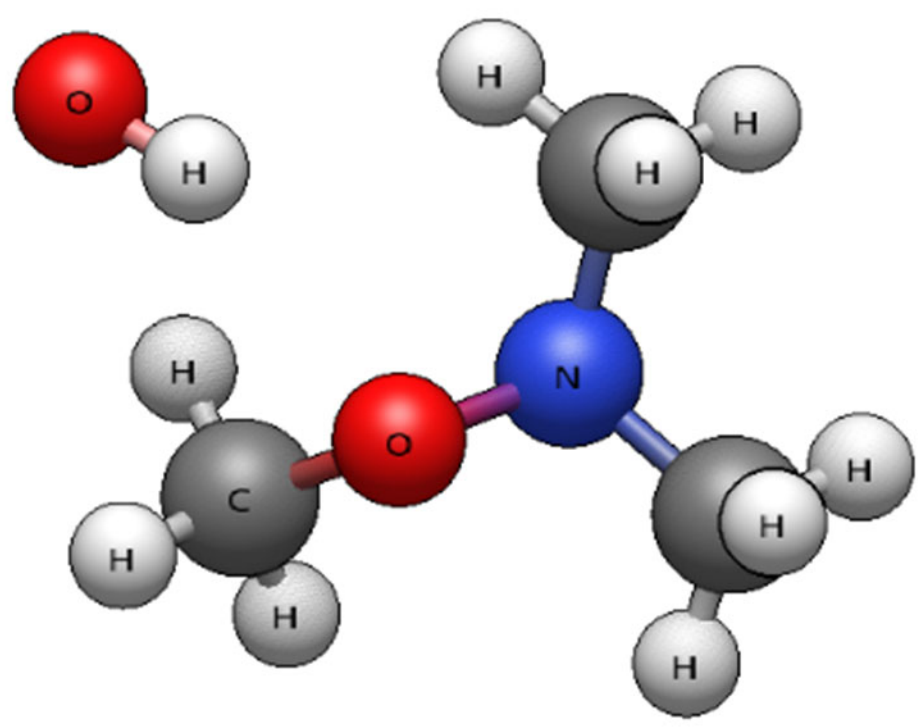


## Cartesian Coordinates

| # | Atom | X (Å) | Y (Å) | Z (Å) |
|---|---|---|---|---|
| 1 | C1 | 0.403640 | 1.202446 | 0.480823 |
| 2 | H2 | 1.339287 | 1.645510 | 0.138423 |
| 3 | H3 | 0.477232 | 1.004716 | 1.560613 |
| 4 | H4 | -0.399079 | 1.915391 | 0.297082 |
| 5 | N5 | 0.161587 | -0.006898 | -0.291626 |
| 6 | C6 | 1.205213 | -1.003975 | -0.092055 |
| 7 | O7 | -1.065954 | -0.575552 | 0.228852 |
| 8 | H8 | -2.302949 | 0.201599 | -1.260352 |
| 9 | H9 | 1.343886 | -1.271383 | 0.965050 |
| 10 | H10 | 0.962941 | -1.903046 | -0.657490 |
| 11 | H11 | 2.139490 | -0.594967 | -0.481168 |
| 12 | H12 | -1.591109 | -1.388436 | -1.627110 |
| 13 | C13 | -1.997060 | -0.758874 | -0.831004 |
| 14 | H14 | -2.858441 | -1.249430 | -0.376958 |
| 15 | O15 | -3.205972 | 1.670333 | 0.512241 |
| 16 | H16 | -2.493494 | 1.121186 | 0.900408 |

## Bond Lengths

| # | Bond | Length |
|---|---|---|
| 1 | C1 – H2 | **1.0904 Å** |
| 2 | C1 – H3 | **1.1002 Å** |
| 3 | C1 – H4 | **1.0892 Å** |
| 4 | C1 – N5 | **1.4553 Å** |
| 5 | N5 – C6 | **1.4571 Å** |
| 6 | N5 – O7 | **1.4495 Å** |
| 7 | C6 – H9 | **1.0992 Å** |
| 8 | C6 – H10 | **1.0894 Å** |
| 9 | C6 – H11 | **1.0916 Å** |
| 10 | O7 – C13 | **1.4226 Å** |
| 11 | H8 – C13 | **1.0956 Å** |
| 12 | H12 – C13 | **1.0931 Å** |
| 13 | C13 – H14 | **1.0903 Å** |
| 14 | O15 – H16 | **0.9797 Å** |

## Bond Angles

B is the vertex atom in each A–B–C triple.

| # | Angle | Value |
|---|---|---|
| 1 | H2 – C1 – H3 | **108.89 °** |
| 2 | H2 – C1 – H4 | **108.27 °** |
| 3 | H2 – C1 – N5 | **108.28 °** |
| 4 | H3 – C1 – H4 | **109.42 °** |
| 5 | H3 – C1 – N5 | **112.50 °** |
| 6 | H4 – C1 – N5 | **109.38 °** |
| 7 | C1 – N5 – C6 | **112.14 °** |
| 8 | C1 – N5 – O7 | **106.04 °** |
| 9 | C6 – N5 – O7 | **106.79 °** |
| 10 | N5 – C6 – H9 | **112.86 °** |
| 11 | N5 – C6 – H10 | **109.53 °** |
| 12 | N5 – C6 – H11 | **107.93 °** |
| 13 | H9 – C6 – H10 | **109.05 °** |
| 14 | H9 – C6 – H11 | **109.03 °** |
| 15 | H10 – C6 – H11 | **108.33 °** |
| 16 | N5 – O7 – C13 | **109.71 °** |

| # | Angle | Value |
|---|---|---|
| 17 | O7 – C13 – H8 | **111.21 °** |
| 18 | O7 – C13 – H12 | **111.95 °** |
| 19 | O7 – C13 – H14 | **105.36 °** |
| 20 | H8 – C13 – H12 | **108.85 °** |
| 21 | H8 – C13 – H14 | **109.70 °** |
| 22 | H12 – C13 – H14 | **109.73 °** |

## Dihedral Angles

IUPAC signed convention, −180° to +180°.

| # | Dihedral | Value |
|---|---|---|
| 1 | H2 – C1 – N5 – C6 | **-62.09 °** |
| 2 | H2 – C1 – N5 – O7 | **-178.29 °** |
| 3 | H3 – C1 – N5 – C6 | **58.31 °** |
| 4 | H3 – C1 – N5 – O7 | **-57.89 °** |
| 5 | H4 – C1 – N5 – C6 | **-179.87 °** |
| 6 | H4 – C1 – N5 – O7 | **63.93 °** |
| 7 | C1 – N5 – C6 – H9 | **-56.13 °** |
| 8 | C1 – N5 – C6 – H10 | **-177.83 °** |
| 9 | C1 – N5 – C6 – H11 | **64.42 °** |
| 10 | O7 – N5 – C6 – H9 | **59.62 °** |
| 11 | O7 – N5 – C6 – H10 | **-62.09 °** |
| 12 | O7 – N5 – C6 – H11 | **-179.84 °** |
| 13 | C1 – N5 – O7 – C13 | **-124.94 °** |
| 14 | C6 – N5 – O7 – C13 | **115.30 °** |
| 15 | N5 – O7 – C13 – H8 | **65.05 °** |
| 16 | N5 – O7 – C13 – H12 | **-56.96 °** |
| 17 | N5 – O7 – C13 – H14 | **-176.17 °** |

# S15. N,N,O-Trimethylhydroxylamine + Hydroxy radical TS

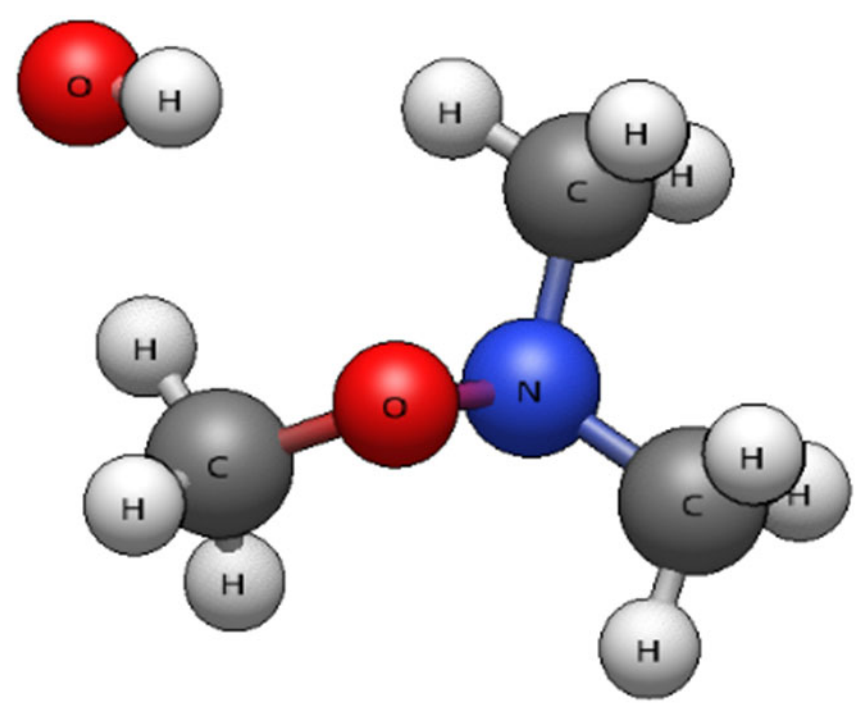


## Cartesian Coordinates

| # | Atom | X (Å) | Y (Å) | Z (Å) |
|---|---|---|---|---|
| 1 | C1 | 0.377009 | 1.184605 | 0.481170 |
| 2 | H2 | 1.279851 | 1.672381 | 0.110068 |
| 3 | H3 | 0.496046 | 0.983850 | 1.555747 |
| 4 | H4 | -0.464466 | 1.858687 | 0.328103 |
| 5 | N5 | 0.170398 | -0.033244 | -0.291885 |
| 6 | C6 | 1.247536 | -0.993861 | -0.102323 |
| 7 | O7 | -1.018068 | -0.671666 | 0.237282 |
| 8 | H8 | -2.487942 | 0.351017 | -0.841613 |
| 9 | H9 | 1.383787 | -1.269960 | 0.952879 |
| 10 | H10 | 1.036346 | -1.892468 | -0.680427 |
| 11 | H11 | 2.170608 | -0.547829 | -0.475914 |
| 12 | H12 | -1.672704 | -0.973765 | -1.712705 |
| 13 | C13 | -2.042645 | -0.658787 | -0.733995 |
| 14 | H14 | -2.813746 | -1.343103 | -0.378667 |
| 15 | O15 | -3.237521 | 1.684602 | 0.404884 |
| 16 | H16 | -2.797863 | 1.158394 | 1.099959 |

## Bond Lengths

| # | Bond | Length |
|---|---|---|
| 1 | C1 – H2 | **1.0912 Å** |
| 2 | C1 – H3 | **1.0996 Å** |

| # | Bond | Length |
|---|---|---|
| 3 | C1 – H4 | **1.0890 Å** |
| 4 | C1 – N5 | **1.4572 Å** |
| 5 | N5 – C6 | **1.4557 Å** |
| 6 | N5 – O7 | **1.4492 Å** |
| 7 | C6 – H9 | **1.0992 Å** |
| 8 | C6 – H10 | **1.0892 Å** |
| 9 | C6 – H11 | **1.0911 Å** |
| 10 | O7 – C13 | **1.4118 Å** |
| 11 | H8 – C13 | **1.1089 Å** |
| 12 | H12 – C13 | **1.0927 Å** |
| 13 | C13 – H14 | **1.0905 Å** |
| 14 | O15 – H16 | **0.9764 Å** |

## Bond Angles

B is the vertex atom in each A–B–C triple.

| # | Angle | Value |
|---|---|---|
| 1 | H2 – C1 – H3 | **108.93 °** |
| 2 | H2 – C1 – H4 | **108.35 °** |
| 3 | H2 – C1 – N5 | **108.09 °** |
| 4 | H3 – C1 – H4 | **109.51 °** |
| 5 | H3 – C1 – N5 | **112.41 °** |
| 6 | H4 – C1 – N5 | **109.46 °** |
| 7 | C1 – N5 – C6 | **112.18 °** |
| 8 | C1 – N5 – O7 | **106.90 °** |
| 9 | C6 – N5 – O7 | **105.58 °** |
| 10 | N5 – C6 – H9 | **112.49 °** |
| 11 | N5 – C6 – H10 | **109.38 °** |
| 12 | N5 – C6 – H11 | **108.16 °** |
| 13 | H9 – C6 – H10 | **109.05 °** |
| 14 | H9 – C6 – H11 | **109.06 °** |
| 15 | H10 – C6 – H11 | **108.64 °** |
| 16 | N5 – O7 – C13 | **109.87 °** |
| 17 | O7 – C13 – H8 | **111.50 °** |
| 18 | O7 – C13 – H12 | **111.58 °** |

| # | Angle | Value |
|---|---|---|
| 19 | O7 – C13 – H14 | **106.46 °** |
| 20 | H8 – C13 – H12 | **108.15 °** |
| 21 | H8 – C13 – H14 | **108.61 °** |
| 22 | H12 – C13 – H14 | **110.51 °** |

## Dihedral Angles

IUPAC signed convention, −180° to +180°.

| # | Dihedral | Value |
|---|---|---|
| 1 | H2 – C1 – N5 – C6 | **-63.69 °** |
| 2 | H2 – C1 – N5 – O7 | **-178.97 °** |
| 3 | H3 – C1 – N5 – C6 | **56.56 °** |
| 4 | H3 – C1 – N5 – O7 | **-58.72 °** |
| 5 | H4 – C1 – N5 – C6 | **178.49 °** |
| 6 | H4 – C1 – N5 – O7 | **63.21 °** |
| 7 | C1 – N5 – C6 – H9 | **-57.37 °** |
| 8 | C1 – N5 – C6 – H10 | **-178.71 °** |
| 9 | C1 – N5 – C6 – H11 | **63.14 °** |
| 10 | O7 – N5 – C6 – H9 | **58.71 °** |
| 11 | O7 – N5 – C6 – H10 | **-62.63 °** |
| 12 | O7 – N5 – C6 – H11 | **179.22 °** |
| 13 | C1 – N5 – O7 – C13 | **-113.68 °** |
| 14 | C6 – N5 – O7 – C13 | **126.69 °** |
| 15 | N5 – O7 – C13 – H8 | **74.63 °** |
| 16 | N5 – O7 – C13 – H12 | **-46.43 °** |
| 17 | N5 – O7 – C13 – H14 | **-167.07 °** |

# S16. N,N-Dimethylaminoxymethyl radical + water

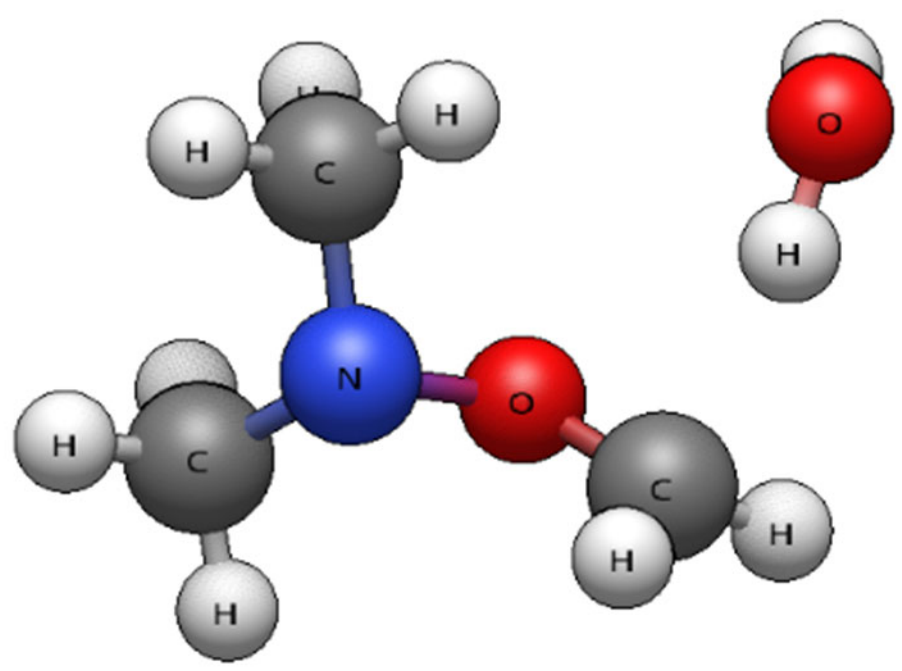


## Cartesian Coordinates

| # | Atom | X (Å) | Y (Å) | Z (Å) |
|---|---|---|---|---|
| 1 | C1 | 0.342494 | 1.148463 | 0.478114 |
| 2 | H2 | 1.206675 | 1.694593 | 0.096334 |
| 3 | H3 | 0.496336 | 0.936948 | 1.545184 |
| 4 | H4 | -0.545772 | 1.765117 | 0.352722 |
| 5 | N5 | 0.208938 | -0.066661 | -0.320258 |
| 6 | C6 | 1.318001 | -0.988699 | -0.120891 |
| 7 | O7 | -0.955392 | -0.777062 | 0.195259 |
| 8 | H8 | -2.915878 | 0.890097 | -0.203307 |
| 9 | H9 | 1.447576 | -1.265972 | 0.933900 |
| 10 | H10 | 1.146782 | -1.887341 | -0.711516 |
| 11 | H11 | 2.226000 | -0.501488 | -0.477669 |
| 12 | H12 | -1.713310 | -0.581115 | -1.744855 |
| 13 | C13 | -1.954140 | -0.819124 | -0.718548 |
| 14 | H14 | -2.756217 | -1.487284 | -0.435985 |
| 15 | O15 | -3.223742 | 1.644127 | 0.334798 |
| 16 | H16 | -3.194316 | 1.304488 | 1.236115 |

## Bond Lengths

| # | Bond | Length |
| --- | --- | --- |
| 1 | C1 – H2 | **1.0912 Å** |
| 2 | C1 – H3 | **1.0987 Å** |
| 3 | C1 – H4 | **1.0886 Å** |
| 4 | C1 – N5 | **1.4601 Å** |
| 5 | N5 – C6 | **1.4560 Å** |
| 6 | N5 – O7 | **1.4581 Å** |
| 7 | C6 – H9 | **1.0983 Å** |
| 8 | C6 – H10 | **1.0889 Å** |
| 9 | C6 – H11 | **1.0905 Å** |
| 10 | O7 – C13 | **1.3544 Å** |
| 11 | H8 – O15 | **0.9762 Å** |
| 12 | H12 – C13 | **1.0807 Å** |
| 13 | C13 – H14 | **1.0815 Å** |
| 14 | O15 – H16 | **0.9636 Å** |

## Bond Angles

B is the vertex atom in each A–B–C triple.

| # | Angle | Value |
| --- | --- | --- |
| 1 | H2 – C1 – H3 | **108.98 °** |
| 2 | H2 – C1 – H4 | **108.81 °** |
| 3 | H2 – C1 – N5 | **107.32 °** |
| 4 | H3 – C1 – H4 | **109.58 °** |
| 5 | H3 – C1 – N5 | **112.56 °** |
| 6 | H4 – C1 – N5 | **109.50 °** |
| 7 | C1 – N5 – C6 | **112.49 °** |
| 8 | C1 – N5 – O7 | **106.57 °** |
| 9 | C6 – N5 – O7 | **104.55 °** |
| 10 | N5 – C6 – H9 | **112.41 °** |
| 11 | N5 – C6 – H10 | **109.18 °** |
| 12 | N5 – C6 – H11 | **107.85 °** |
| 13 | H9 – C6 – H10 | **109.34 °** |
| 14 | H9 – C6 – H11 | **109.19 °** |
| 15 | H10 – C6 – H11 | **108.80 °** |
| 16 | N5 – O7 – C13 | **111.43 °** |

| # | Angle | Value |
|---|---|---|
| 17 | O7 – C13 – H12 | **118.01 °** |
| 18 | O7 – C13 – H14 | **112.94 °** |
| 19 | H12 – C13 – H14 | **123.33 °** |
| 20 | H8 – O15 – H16 | **103.52 °** |

## Dihedral Angles

IUPAC signed convention, −180° to +180°.

| # | Dihedral | Value |
|---|---|---|
| 1 | H2 – C1 – N5 – C6 | **-66.46 °** |
| 2 | H2 – C1 – N5 – O7 | **179.52 °** |
| 3 | H3 – C1 – N5 – C6 | **53.43 °** |
| 4 | H3 – C1 – N5 – O7 | **-60.58 °** |
| 5 | H4 – C1 – N5 – C6 | **175.59 °** |
| 6 | H4 – C1 – N5 – O7 | **61.57 °** |
| 7 | C1 – N5 – C6 – H9 | **-56.48 °** |
| 8 | C1 – N5 – C6 – H10 | **-178.00 °** |
| 9 | C1 – N5 – C6 – H11 | **63.94 °** |
| 10 | O7 – N5 – C6 – H9 | **58.75 °** |
| 11 | O7 – N5 – C6 – H10 | **-62.76 °** |
| 12 | O7 – N5 – C6 – H11 | **179.18 °** |
| 13 | C1 – N5 – O7 – C13 | **-113.66 °** |
| 14 | C6 – N5 – O7 – C13 | **127.03 °** |
| 15 | N5 – O7 – C13 – H12 | **-15.33 °** |
| 16 | N5 – O7 – C13 – H14 | **-169.61 °** |

# S17. N,N-Dimethylaminoxymethyl radical fragmentation TS

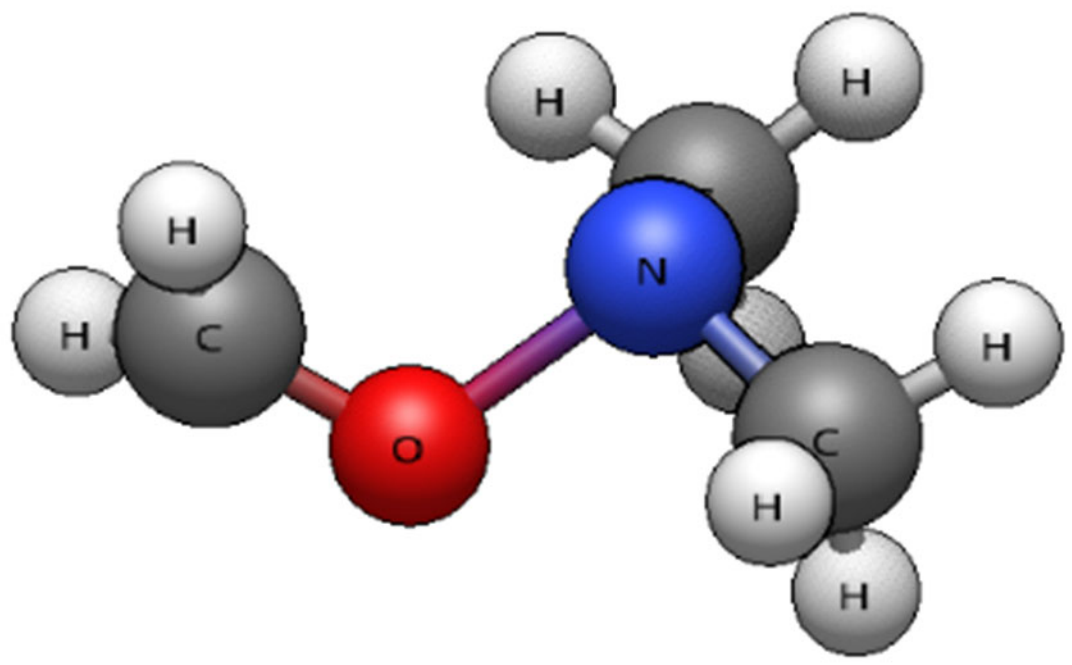


## Cartesian Coordinates

| # | Atom | X (Å) | Y (Å) | Z (Å) |
|---|---|---|---|---|
| 1 | C1 | 1.381723 | 1.067161 | -0.017798 |
| 2 | H2 | 2.342349 | 0.904158 | -0.516833 |
| 3 | H3 | 1.549609 | 1.075893 | 1.067446 |
| 4 | H4 | 0.991140 | 2.033928 | -0.331836 |
| 5 | N5 | 0.481704 | 0.019251 | -0.446363 |
| 6 | C6 | 0.846165 | -1.278359 | 0.080636 |
| 7 | O7 | -0.904091 | 0.396138 | 0.455308 |
| 8 | H8 | -2.693194 | -0.599979 | 0.344757 |
| 9 | H9 | 1.070857 | -1.267433 | 1.155546 |
| 10 | H10 | 0.043900 | -1.990159 | -0.117528 |
| 11 | H11 | 1.735525 | -1.619289 | -0.461060 |
| 12 | H12 | -2.186769 | 0.420842 | -1.163142 |
| 13 | C13 | -2.001175 | 0.083300 | -0.145514 |

## Bond Lengths

| # | Bond | Length |
|---|---|---|
| 1 | C1 – H2 | **1.0947 Å** |
| 2 | C1 – H3 | **1.0982 Å** |
| 3 | C1 – H4 | **1.0890 Å** |
| 4 | C1 – N5 | **1.4463 Å** |

| # | Bond | Length |
|---|---|---|
| 5 | N5 – C6 | **1.4472 Å** |
| 6 | N5 – O7 | **1.6957 Å** |
| 7 | C6 – H9 | **1.0982 Å** |
| 8 | C6 – H10 | **1.0907 Å** |
| 9 | C6 – H11 | **1.0957 Å** |
| 10 | O7 – C13 | **1.2894 Å** |
| 11 | H8 – C13 | **1.0891 Å** |
| 12 | H12 – C13 | **1.0881 Å** |

## Bond Angles

B is the vertex atom in each A–B–C triple.

| # | Angle | Value |
|---|---|---|
| 1 | H2 – C1 – H3 | **108.51 °** |
| 2 | H2 – C1 – H4 | **108.39 °** |
| 3 | H2 – C1 – N5 | **107.64 °** |
| 4 | H3 – C1 – H4 | **109.44 °** |
| 5 | H3 – C1 – N5 | **113.19 °** |
| 6 | H4 – C1 – N5 | **109.55 °** |
| 7 | C1 – N5 – C6 | **112.65 °** |
| 8 | C1 – N5 – O7 | **100.95 °** |
| 9 | C6 – N5 – O7 | **102.21 °** |
| 10 | N5 – C6 – H9 | **113.52 °** |
| 11 | N5 – C6 – H10 | **109.50 °** |
| 12 | N5 – C6 – H11 | **107.66 °** |
| 13 | H9 – C6 – H10 | **109.56 °** |
| 14 | H9 – C6 – H11 | **108.72 °** |
| 15 | H10 – C6 – H11 | **107.71 °** |
| 16 | N5 – O7 – C13 | **113.18 °** |
| 17 | O7 – C13 – H8 | **118.89 °** |
| 18 | O7 – C13 – H12 | **120.38 °** |
| 19 | H8 – C13 – H12 | **120.48 °** |

## Dihedral Angles

IUPAC signed convention, −180° to +180°.

| # | Dihedral | Value |
|---|---|---|
| 1 | H2 – C1 – N5 – C6 | **-69.69 °** |
| 2 | H2 – C1 – N5 – O7 | **-178.00 °** |
| 3 | H3 – C1 – N5 – C6 | **50.22 °** |
| 4 | H3 – C1 – N5 – O7 | **-58.09 °** |
| 5 | H4 – C1 – N5 – C6 | **172.65 °** |
| 6 | H4 – C1 – N5 – O7 | **64.34 °** |
| 7 | C1 – N5 – C6 – H9 | **-46.90 °** |
| 8 | C1 – N5 – C6 – H10 | **-169.68 °** |
| 9 | C1 – N5 – C6 – H11 | **73.49 °** |
| 10 | O7 – N5 – C6 – H9 | **60.61 °** |
| 11 | O7 – N5 – C6 – H10 | **-62.18 °** |
| 12 | O7 – N5 – C6 – H11 | **-179.00 °** |
| 13 | C1 – N5 – O7 – C13 | **-148.39 °** |
| 14 | C6 – N5 – O7 – C13 | **95.31 °** |
| 15 | N5 – O7 – C13 – H8 | **-122.21 °** |
| 16 | N5 – O7 – C13 – H12 | **52.02 °** |

# S18. Dimethylamino radical

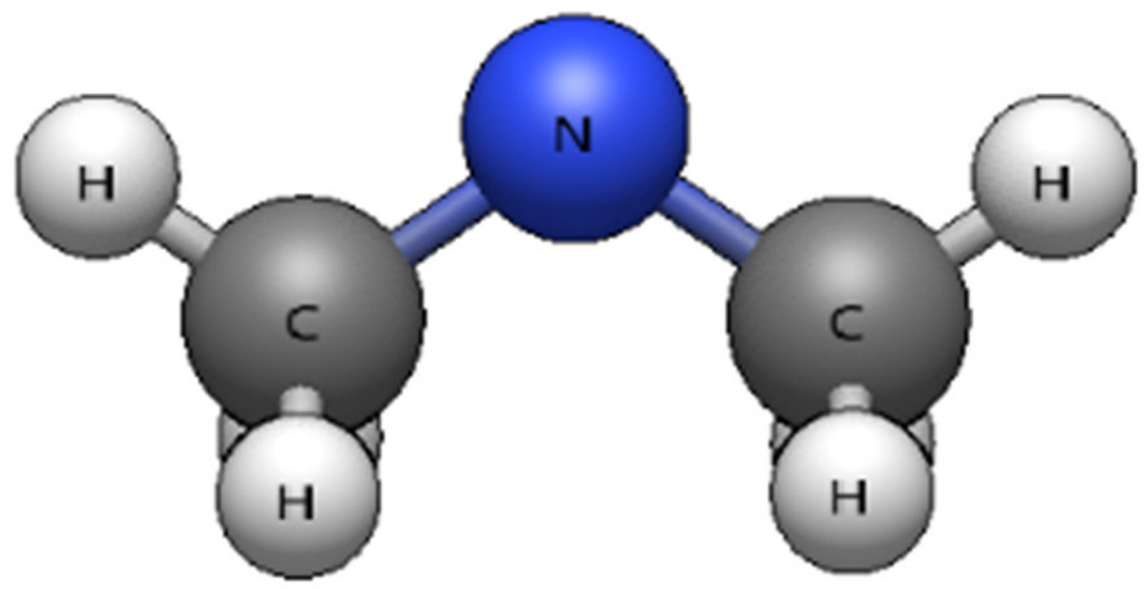


## Cartesian Coordinates

| # | Atom | X (Å) | Y (Å) | Z (Å) |
|---|---|---|---|---|
| 1 | C1 | 1.291706 | 1.070379 | -0.022567 |
| 2 | H2 | 2.237089 | 0.911072 | -0.560766 |
| 3 | H3 | 1.537827 | 1.088033 | 1.048962 |
| 4 | H4 | 0.881008 | 2.038275 | -0.306345 |
| 5 | N5 | 0.347550 | 0.015333 | -0.313624 |
| 6 | C6 | 0.907711 | -1.267477 | 0.046777 |
| 7 | H7 | 1.176424 | -1.293283 | 1.112660 |
| 8 | H8 | 0.195637 | -2.065494 | -0.158552 |
| 9 | H9 | 1.832053 | -1.460933 | -0.516324 |

## Bond Lengths

| # | Bond | Length |
|---|---|---|
| 1 | C1 – H2 | **1.0994 Å** |
| 2 | C1 – H3 | **1.0996 Å** |
| 3 | C1 – H4 | **1.0890 Å** |
| 4 | C1 – N5 | **1.4454 Å** |
| 5 | N5 – C6 | **1.4454 Å** |
| 6 | C6 – H7 | **1.0995 Å** |
| 7 | C6 – H8 | **1.0891 Å** |
| 8 | C6 – H9 | **1.0995 Å** |

## Bond Angles

B is the vertex atom in each A–B–C triple.

| # | Angle | Value |
|---|---|---|
| 1 | H2 – C1 – H3 | **106.67 °** |
| 2 | H2 – C1 – H4 | **109.00 °** |
| 3 | H2 – C1 – N5 | **110.94 °** |
| 4 | H3 – C1 – H4 | **108.91 °** |
| 5 | H3 – C1 – N5 | **110.74 °** |
| 6 | H4 – C1 – N5 | **110.48 °** |
| 7 | C1 – N5 – C6 | **110.15 °** |
| 8 | N5 – C6 – H7 | **110.93 °** |
| 9 | N5 – C6 – H8 | **110.48 °** |
| 10 | N5 – C6 – H9 | **110.75 °** |
| 11 | H7 – C6 – H8 | **108.99 °** |
| 12 | H7 – C6 – H9 | **106.67 °** |
| 13 | H8 – C6 – H9 | **108.92 °** |

## Dihedral Angles

IUPAC signed convention, −180° to +180°.

| # | Dihedral | Value |
|---|---|---|
| 1 | H2 – C1 – N5 – C6 | **-58.14 °** |
| 2 | H3 – C1 – N5 – C6 | **60.11 °** |
| 3 | H4 – C1 – N5 – C6 | **-179.13 °** |
| 4 | C1 – N5 – C6 – H7 | **-58.30 °** |
| 5 | C1 – N5 – C6 – H8 | **-179.28 °** |
| 6 | C1 – N5 – C6 – H9 | **59.95 °** |

# S19. Formaldehyde

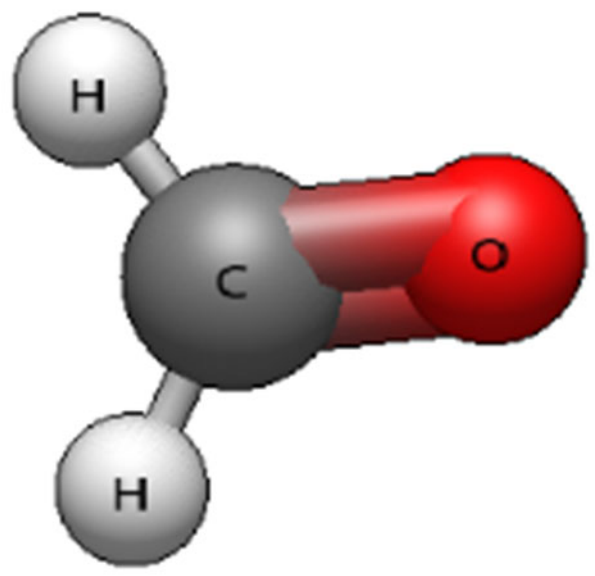


## Cartesian Coordinates

| # | Atom | X (Å) | Y (Å) | Z (Å) |
|---|---|---|---|---|
| 1 | O1 | -0.805874 | 0.178761 | 0.431979 |
| 2 | H2 | -2.824991 | 0.147029 | 0.317496 |
| 3 | H3 | -1.863526 | 0.042661 | -1.286691 |
| 4 | C4 | -1.843844 | 0.122152 | -0.186453 |

## Bond Lengths

| # | Bond | Length |
|---|---|---|
| 1 | O1 – C4 | **1.2096 Å** |
| 2 | H2 – C4 | **1.1033 Å** |
| 3 | H3 – C4 | **1.1033 Å** |

## Bond Angles

B is the vertex atom in each A–B–C triple.

| # | Angle | Value |
|---|---|---|
| 1 | O1 – C4 – H2 | **121.91 °** |
| 2 | O1 – C4 – H3 | **121.91 °** |
| 3 | H2 – C4 – H3 | **116.19 °** |

## Dihedral Angles

No dihedral angles found.

**Table S20. Focal point table of (CH3)2NOCH2• + H with respect to (CH3)2NOCH3.**

| | E_HF | +δE_MP2 | +δE_CCSD | +δE_CCSD(T) | +δE_T | NET |
|---|---|---|---|---|---|---|
| DZ | +83.88 | +14.63 | +0.64 | +0.18 | –0.07 | [+99.26] |
| TZ | +83.21 | +18.97 | +0.22 | +0.26 | [–0.07] | [+102.58] |
| QZ | +83.21 | +19.93 | +0.01 | +0.28 | [–0.07] | [+103.35] |
| 5Z | +83.21 | +20.21 | –0.10 | +0.27 | [–0.07] | [+103.52] |
| CBS | [+83.21] | [+20.50] | [–0.22] | [+0.27] | [–0.07] | **[+103.70]** |

*ΔE = δE_CCSD(T)/CBS + δE_ZPVE + δE_aZPVE*

$\Delta E = 103.70 - 8.64 + 0.07 = 95.13\ kcal\ mol^{-1}$

**Table S21. Focal point table of (CH3)(CH2•)NOCH3 + H with respect to (CH3)2NOCH3.**

| | E_HF | +δE_MP2 | +δE_CCSD | +δE_CCSD(T) | +δE_T | NET |
|---|---|---|---|---|---|---|
| DZ | +83.81 | +15.61 | +0.16 | +0.34 | +0.01 | [+99.52] |
| TZ | +83.15 | +19.35 | –0.24 | +0.37 | [+0.01] | [+102.64] |
| QZ | +83.08 | +20.15 | –0.47 | +0.37 | [+0.01] | [+103.14] |
| 5Z | +83.04 | +20.39 | –0.58 | +0.35 | [+0.01] | [+103.21] |
| CBS | [+83.02] | [+20.64] | [–0.70] | [+0.34] | [+0.01] | **[+103.30]** |

*ΔE = δE_CCSD(T)/CBS + δE_ZPVE + δE_aZPVE*

$\Delta E = 103.30 - 8.68 - 0.25 = 94.37\ kcal\ mol^{-1}$

**Table S22. Focal point table of methoxymethyl radical + H with respect to dimethyl ether.**

| | E_HF | +δE_MP2 | +δE_CCSD | +δE_CCSD(T) | +δE_T | +δE_(Q) | NET |
|---|---|---|---|---|---|---|---|
| DZ | +82.02 | +15.95 | +0.65 | +0.25 | –0.42 | +0.01 | [+98.46] |
| TZ | +82.30 | +19.48 | +0.21 | +0.35 | [–0.42] | [+0.01] | [+101.92] |
| QZ | +82.60 | +20.15 | –0.00 | +0.37 | [–0.42] | [+0.01] | [+102.71] |
| 5Z | +82.59 | +20.45 | –0.11 | +0.37 | [–0.42] | [+0.01] | [+102.88] |
| CBS | [+82.55] | [+20.75] | [–0.22] | [+0.37] | [–0.42] | [+0.01] | **[+103.03]** |

*ΔE = δE_CCSD(T)/CBS + δE_ZPVE + δE_aZPVE*

$\Delta E = 103.03 - 8.71 + 0.02 = 94.34\ kcal\ mol^{-1}$

**Table S23. Focal point table of dimethylaminomethyl radical + H with respect to trimethylamine.**

| | E_HF | +δE_MP2 | +δE_CCSD | +δE_CCSD(T) | +δE_T | NET |
|---|---|---|---|---|---|---|
| DZ | +79.42 | +16.25 | +0.48 | +0.33 | +0.02 | [+96.50] |
| TZ | +77.87 | +20.63 | +0.12 | +0.37 | [+0.02] | [+99.01] |
| QZ | +79.74 | +19.39 | −0.11 | +0.34 | [+0.02] | [+99.39] |
| 5Z | +79.69 | +19.60 | −0.21 | +0.32 | [+0.02] | [+99.41] |
| CBS | [+79.49] | [+19.82] | [−0.32] | [+0.29] | [+0.02] | **[+99.29]** |

*ΔE = δE_CCSD(T)/CBS + δE_ZPVE + δE_aZPVE*

*ΔE = 99.29 − 8.41 + 1.00 = 91.88 kcal mol⁻¹*

**Table S24. Focal point table of dimethylaminyl radical + formaldehyde with respect to (CH3)2NOCH2•.**

| | E_HF | +δE_MP2 | +δE_CCSD | +δE_CCSD(T) | +δE_T | NET |
|---|---|---|---|---|---|---|
| DZ | −34.74 | +17.38 | −3.25 | +1.43 | −1.10 | [−20.28] |
| TZ | −31.78 | +15.34 | −3.79 | +1.71 | [−1.10] | [−19.62] |
| QZ | −31.85 | +16.16 | −3.92 | +1.82 | [−1.10] | [−18.89] |
| 5Z | −31.97 | +16.40 | −3.90 | +1.87 | [−1.10] | [−18.70] |
| CBS | [−32.05] | [+16.65] | [−3.89] | [+1.92] | [−1.10] | **[−18.46]** |

*ΔE = δE_CCSD(T)/CBS + δE_ZPVE + δE_aZPVE*

*ΔE = −18.46 − 3.95 + 0.07 = −22.34 kcal mol⁻¹*

**Table S25. Focal point table of N-Methylmethanimine + methoxyl radical with respect to (CH3)(CH2•)NOCH3.**

| | E_HF | +δE_MP2 | +δE_CCSD | +δE_CCSD(T) | +δE_T | NET |
|---|---|---|---|---|---|---|
| DZ | −27.63 | +23.18 | −4.78 | +1.33 | −1.07 | [−8.97] |
| TZ | −23.68 | +21.49 | −6.19 | +1.77 | [−1.07] | [−7.67] |
| QZ | −24.37 | +22.82 | −6.40 | +1.92 | [−1.07] | [−7.11] |
| 5Z | −24.42 | +23.18 | −6.46 | +1.97 | [−1.07] | [−6.79] |
| CBS | [−24.38] | [+23.57] | [−6.52] | [+2.03] | [−1.07] | **[−6.36]** |

*ΔE = δE_CCSD(T)/CBS + δE_ZPVE*

*ΔE = −6.36 − 3.35 = −9.71 kcal mol⁻¹*
